# Phenotypic and Functional Plasticity of CXCR6^+^ Peripheral Blood NK Cells

**DOI:** 10.3389/fimmu.2021.810080

**Published:** 2022-01-31

**Authors:** Laura S. Angelo, Graham D. Hogg, Shawn Abeynaike, Lynn Bimler, Alexander Vargas-Hernandez, Silke Paust

**Affiliations:** ^1^ Center for Human Immunobiology, Department of Pediatrics, Texas Children’s Hospital, Houston, TX, United States; ^2^ Department of Immunology and Microbiology, The Scripps Research Institute, La Jolla, CA, United States

**Keywords:** NK cell, CXCR6, tissue resident NK cells, peripheral blood NK cells, phenotypic and functional plasticity

## Abstract

Human NK cells are comprised of phenotypic subsets, whose potentially unique functions remain largely unexplored. C-X-C-motif-chemokine-receptor-6 (CXCR6)**^+^** NK cells have been identified as phenotypically immature tissue-resident NK cells in mice and humans. A small fraction of peripheral blood (PB)-NK cells also expresses CXCR6. However, prior reports about their phenotypic and functional plasticity are conflicting. In this study, we isolated, expanded, and phenotypically and functionally evaluated CXCR6^+^ and CXCR6^–^ PB-NK cells, and contrasted results to bulk liver and spleen NK cells. We found that CXCR6^+^ and CXCR6^–^ PB-NK cells preserved their distinct phenotypic profiles throughout 14 days of *in vitro* expansion (“day 14”), after which phenotypically immature CXCR6^+^ PB-NK cells became functionally equivalent to CXCR6^–^ PB-NK cells. Despite a consistent reduction in CD16 expression and enhanced expression of the transcription factor Eomesodermin (Eomes), day 14 CXCR6^+^ PB-NK cells had superior antibody-dependent cellular cytotoxicity (ADCC) compared to CXCR6^–^ PB-NK cells. Further, bulk liver NK cells responded to IL-15, but not IL-2 stimulation, with STAT-5 phosphorylation. In contrast, bulk splenic and PB-NK cells robustly responded to both cytokines. Our findings may allow for the selection of superior NK cell subsets for infusion products increasingly used to treat human diseases.

## Introduction

Natural Killer (NK) cells are cytotoxic lymphocytes indispensable for human health (1). NK cells use a polymorphic repertoire of activating and inhibitory receptors to discriminate healthy from infected or transformed target cells (2, 3). NK cells rapidly kill their targets by perforin and granzyme release without the need for prior activation (4) (5). NK cell secreted cytokines and their effector functions modulate adaptive immune responses (6). Similarly, costimulatory receptors and cytokine and chemokine signaling modulate NK cell development and function (7). Important cytokines for NK cell development and effector functions include the common gamma chain-dependent cytokines IL-2 and IL-15 (8). Their receptors activate Janus kinase (JAK) and signal transducer and activator of transcription (STAT) pathways, leading to gene transcription, additional NK cell differentiation, and/or modulation of effector functions. Due to their diverse and robust immune capabilities, NK cells are widely used for immunotherapy (9). Despite a significant increase in NK cell-based clinical applications, including the infusion of NK cell products into humans, many essential aspects of NK cell biology remain poorly understood.

Human NK cells are found in lymphoid and non-lymphoid tissues and comprise many phenotypic subsets whose potentially unique functions remain unexplored. In humans, NK cells make up 5-20% of immune cells in PB and secondary lymphoid tissues (10). NK cells are even more abundant in non-lymphoid tissues, comprising 30-80% of all immune cells depending on tissue origin. Most easily accessible and extensively studied are PB-NK cells, generally mature in phenotype (CD56^lo^ CD16^+^) and highly cytotoxic. However, this phenotype and functional capability does not reflect that of tissue-resident NK cells, which are abundant and generally immature in phenotype (CD56^hi^ CD16^–^), express CXCR6 and CD69, and are associated with increased cytokine production and reduced killing capacity (11–17). Tissue-resident NK cells also play an essential role in maintaining the immunotolerant environment in multiple organs (10, 16–20), and their uniqueness has drawn considerable research interest (11–14, 16–19, 21).

NK cell subset- and tissue-specific expression of transcription factors govern NK cell development (22). T-box expressed in T cells (T-bet), and Eomes are two T-box transcription factors that govern later stages of NK cell differentiation. PB-NK cells preferentially express T-bet over Eomes, while tissue-resident NK cells are generally Eomes positive (14, 22). Several reports, including ours, have established that CD56^hi^ CXCR6^+^ Eomes^+^ NK cells are predominantly tissue-resident, while only a minute proportion (2-5%) of PB-NK cells express CXCR6 (11, 14, 17). While both CD56^lo^ and CD56^hi^ PB-NK cells express CXCR6, a higher proportion of CXCR6^+^ NK cells are CD56 ^hi^. The phenotype of this small percentage of CXCR6^+^ PB-NK cells is strikingly like NK cells found in secondary lymphoid organs (CD56^hi^ CXCR6^+^ CD16^lo^, CD69^+^ Eomes^+^) (11, 14, 16–18). This distinct tissue-resident NK cell phenotype indicates the potential for specialized functions unique from those performed by PB-NK cells, which are generally CD56^lo^ CXCR6^–^ T-bet^+^ (16, 18, 19).

CXCR6 is a G-protein-coupled receptor with seven transmembrane domains whose exclusive ligand is C-X-C-motif-chemokine-ligand-16 (CXCL16). CXCL16, also a transmembrane protein, is expressed constitutively on liver sinusoidal endothelial cells, in the spleen red pulp, intestine, lungs, and skin (23). In mice, CXCR6-dependent signaling is essential for the survival of CXCR6^+^, but not CXCR6^–^ hepatic NK cells, and murine and human CXCR6^+^ NK cells are enriched in the liver and other non-lymphoid tissues (18, 23–25). Interestingly, we previously demonstrated that lung and liver derived CXCR6^+^ NK cells mediate immunological memory to viral antigens and inactivated viruses in mice (24, 25). In agreement with these findings, we also recently published that human CXCR6^+^ NK cells with a tissue-resident phenotype (CD56^hi^ CD69^+^ CXCR6^+^ Eomes^hi^) accumulate at sites of antigen-rechallenge and mediate antigen-specific killing *in vitro* (26). However, phenotypic and functional differences between CXCR6^+^ and CXCR6^–^ NK cells from human PB, liver, and spleen have not been thoroughly explored.

## Methods

### PBMC and NK Cell Isolation

PB-NK cells were harvested from healthy adult volunteers (lab and healthcare workers in the Texas Medical Center), or purchased from the Gulf Coast Regional Blood Center for more extensive experiments. Volunteers are not age or sex matched. Blood was treated with NK Rosette Sep (Stem Cell Technologies) as per manufacturer’s instructions then separated by density centrifugation as described (13). Cells were washed twice, counted, and either further enriched for NK cells before sorting, sorted directly, or used as controls for flow cytometry or ^51^Cr release assays. For total PBMC isolation, peripheral blood was immediately diluted 1:1 with phosphate buffered saline (PBS) and layered over Ficoll-Pacque PLUS (GE Healthcare), centrifuged, washed, and counted for use as controls.

### Adult Liver and Spleen Samples

All healthy human liver perfusates were obtained from cadaver donors, centrifuged, resuspended in RPMI, and then separated by density centrifugation. Cells were washed in PBS, counted, and used immediately for ^51^Cr assays or multiparameter flow cytometry. Adult spleen tissue was obtained from patients undergoing PDAC surgery and processed as described (14).

### Cell Line Maintenance

K562, Raji, and HEK293T cell lines were purchased from ATCC. The RPMI-8866 B cell line (Sigma) was used as a feeder cell line for CXCR6^+^ and CXCR6^–^ NK cell cultures. K562, Raji, and RPMI-8866 were maintained in RPMI-1640 (HyClone Laboratories) + 10% fetal bovine serum (FBS), L-glutamine, and penicillin/streptomycin (R10 media) at 37°C, 5% CO_2_. HEK293T cells were maintained in Dulbecco’s Modified Eagle’s Medium (DMEM) plus 10% FBS, L-glutamine, and penicillin/streptomycin. All cell lines were mycoplasma negative.

### Preparation of 96-Well Plates Containing Feeder Cells for CXCR6^+^ and CXCR6^–^ NK Cell Cultures

Because CXCR6^+^ NK cells depend on CXCL16 expression for survival in the liver sinusoidal epithelium (18, 25), we transduced a B lymphoblastoid cell line (RPMI-8866) with lentiviral particles (pLenti-C-mGFP, Origene) encoding either CXCL16 transcript variant 1 (TV1, NM_022059) or 2 (TV2, NM_001100812), thereby generating B cell lines stably expressing the ligand for CXCR6. Lentivirus particles were amplified by infecting HEK293T cells, harvesting the supernatant, and infecting 8866 cells. After expanding the cells, they were sorted/purified based on GFP expression using a FACSAria II (Becton Dickinson). RPMI-8866 cells did not express CXCR6 and were highly positive for CXCL16 following transduction (**Supplementary Figure 1C**). Allogeneic cryopreserved PBMCs were used as additional feeder cells to stimulate NK cell growth in combination with RPMI-8866-CXCL16 cells at a ratio of 2 PBMC to 1 8866-CXCL16. Cells were treated with 10 μg/mL Mitomycin C (Sigma) for 2 hours at 37°C, followed by 10,000 rads of X-ray irradiation (**Supplementary Figure 7**). Cells were washed three times and resuspended in RPMI + 10% human AB serum (Sigma), IL-2 (100 IU/mL, PeproTech), IL-15 (10 ng/mL, Biolegend), IL-15R-Fc chimera (20 ng/mL, R&D Systems), and PHA (2 μg/mL) and plated into 96-well plates. CXCR6^-^ or CXCR6^+^ NK cells were sorted into the wells containing the feeder cells, and 50 μL of media was added immediately after sorting.

### Sorting and Short-Term *Ex Vivo* Culture of CXCR6^-^ and CXCR6^+^ NK Cells for Phenotypic and Functional Studies

To study phenotypic and functional differences between CXCR6^-^ and CXCR6^+^ NK cells, NK cells were enriched from PB using Rosette Sep and human NK cell negative selection columns (Miltenyi). Cells were stained for sorting on a FACSAria II using the antibodies listed in **Supplementary Table 2**. LIVE/DEAD fixable blue dead cell stain kit (Life Technologies) was included as a viability stain. Gating strategy and post-sort purity are shown in **Supplementary Figure 1**. CXCR6^-^ and CXCR6^+^ NK cells were sorted into separate 96-well plates seeded with feeder cells and stimulation media ranging from 100-1,000 cells per well, depending on % CXCR6^+^ NK cells in each donor. After sorting, plates were incubated at 37°C, 5% CO_2_ and expanded into 24, then six-well plates (Corning). Cultures were harvested on day 14 for phenotypic and functional assays.

### Chromium Release Assays

NK cell cytotoxic function was evaluated by ^51^Cr-release assays using K562 erythroleukemia cells or Raji B lymphoma cells as targets for cytotoxicity and ADCC, respectively [as described (14)]. Briefly, CXCR6^+^ and CXCR6^–^ NK cell cultures were harvested on day 14, counted, and viability assessed. Fresh bulk PBMCs from healthy donors were used as controls for both assays. Bulk liver (mean 42% NK cells) and spleen (mean 11.4% NK cells) cells were used to determine NK cell cytotoxic function in these tissues. Initial effector to target cell ratios (E:T) depended on NK cell numbers available in the cultures. Spontaneous release of ^51^Cr into the supernatant by target cells alone was used as background and subtracted from all experimental values (cpm). Percent specific lysis was calculated as described (14).

### Degranulation Assays and Intracellular Flow Cytometry (ICFC)

Mediators of NK cell cytotoxic activity and degranulation (perforin, Granzyme B, CD107a) (27, 28) and activating cytokines (TNFα, IFNγ), were measured using ICFC. Experiments were performed as described (14). Cells were stained with extracellular antibodies (**Supplementary Table 3**), washed, permeabilized, and then immuno-stained with intracellular antibodies (**Supplementary Table 3**). Cells were kept at 4°C in the dark until acquisition (BD LSRII Fortessa with 18 color configuration).

### Phenotyping of CXCR6^+^ and CXCR6^–^ NK Cells by Flow Cytometry or CyTOF

To determine phenotypic differences or changes in CXCR6^+^ and CXCR6^–^ NK cells in PB, liver, and spleen, cells were freshly stained *ex vivo*. Staining of extracellular and intracellular markers was performed as described previously (14). Two separate flow panels were employed (**Supplementary Tables 4, 5**). CyTOF staining and analysis of *in vitro* expanded PB-NK cell subsets was performed as described (26).

### Phosphorylation of STAT5 by Flow Cytometry

In order to determine if NK cells in liver and spleen are capable of being activated by IL-2 and/or IL-15 we thawed cryopreserved PBMCS, liver, and spleen cells and rested them for one hour in R10 media. Cells were washed with PBS, stained with viability dye, and then immuno-stained for surface markers (**Supplementary Table 7**). Cells were then stimulated with 10 ng/mL recombinant human IL-2 (Peprotech) or 50 ng/mL IL-15 (Peprotech) for 1 hour at 37°C, and then fixed with BD Cytofix for 10 minutes at RT. After washing, cells were permeabilized using BD Perm III buffer for 30 minutes on ice, washed, and stained for p-STAT5(Y694) for 45 minutes at 4-8°C. Cells were washed after staining, resuspended in 1% PFA, and stored at 4°C until acquisition.

### Data Analysis and Software

All samples were acquired on a BD LSRII Fortessa. FCS files were transferred to FlowJo software Version 10.1 (TreeStar, Inc). The gating strategy is as shown in **Supplementary Figures 2–5**. Fluorescence minus one (FMO) stains were used in every experiment to determine positive and negative populations, and compensation was performed individually for each experiment using OneComp eBeads (eBioscience). Gates were applied from FMOs to samples, and values for MFI and percent positive cells were transferred to Graph Pad Prism for statistical analysis. Analysis of CyTOF data was initially performed using FlowJo for hierarchical gating of live CXCR6^+^ and CXCR6^-^ NK cells and FCS files exported. FCS files were then analyzed, and figures produced using the cytofkit package (29) in R (R Core team 2021).

### Statistical Analysis

Graphs show data points representing individual flow cytometry samples. Mean and standard deviation for each marker are shown. Statistical analysis was performed using Prism 7 (GraphPad). A student’s t-test with Welch’s correction was utilized to compare unpaired sample groups (liver vs. PB, etc.). However, paired samples (organs or NK cell subsets from the same individual) were analyzed with a paired Student’s t-test. A one-way ANOVA was employed for comparisons of 3 or more groups, accompanied by a multiple comparisons correction. If comparing a single control group to multiple experimental groups, either a Dunnett’s or Holm-Sidak’s multiple comparison test was utilized. If several groups were being compared together, a Tukey’s correction was employed. If the multiple groups were being compared across different conditions, a two-way ANOVA was used. A p-value of less than 0.05 was considered statistically significant and p values were represented as *p < 0.05, **p < 0.01, ***p < 0.001, and ****p < 0.0001.

### Study Approval

All human PB, liver, and spleen tissues were obtained with written informed consent, and the protocols were approved for use by the NIH and by BCM’s and TSRI’s Institutional Review Boards for the Protection of Human Subjects, and in accordance with the Declaration of Helsinki.

## Results

### CXCR6^+^ and CXCR6^–^ NK Cells Differ in Their Expansion Capacity and Phenotypic Profiles

**Figure 1:**
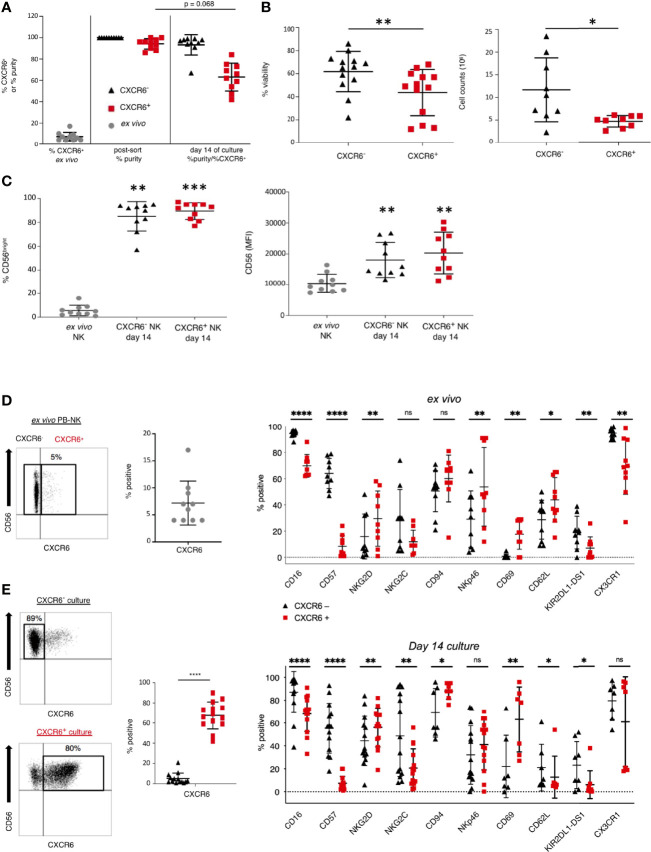
CXCR6- and + NK cell cultures differ in purity, viability, and expansion capacity after *in vitro* expansion. **(A)** Percent CXCR6^+^ NK cells in pre-sorted PB and purity of CXCR6^-^ and + NK cell cultures immediately post-sort and after a 14 day *in vitro* expansion. Pre-sorted *ex vivo* NK cells (gray circles), CXCR6^-^ NK (black triangles), and CXCR6^+^ NK (red squares). All values are mean +/– standard deviation (*n=10)*. **(B)** CXCR6^-^ NK cells are more viable and expand more readily than CXCR6^+^ NK cells at day 14 of culture (*n=13*). Cells were counted using a hemocytometer and trypan blue exclusion (*n=9*). **(C)** CD56^bright^ NK cells increase in CXCR6^-^ and + NK cell cultures by day 14. Percent CD56^+^ and CD56 MFI increases in both subsets compared to fresh donor-matched NK cells (*n=10*). One-way ANOVA with Dunn’s multiple comparisons test **(A, C)** and Wilcoxon matched-pairs signed-rank test were performed **(B)**. **(D)** CXCR6- and + PB-NK cells have distinct phenotypic profiles *ex vivo* (*n=9-10*), which are enhanced after *in vitro* expansion (*n=7–14*) **(E)**. CXCR6^-^ NK cells within CXCR6^-^ 14-day cultures were gated, and CXCR6^+^ NK cells within CXCR6^+^ NK cell cultures were gated for analysis following immuno-staining and flow cytometry. Paired t-tests were performed for each marker. (*p < 0.05; **p < 0.01; ***p < 0.001; ****p < 0.0001; ns, not significant).

We sorted CXCR6^+^ and CXCR6^–^ PB-NK cells from healthy donors (**Figure 1A**) according to the gating strategy shown in **Supplementary Figure 1A**. NK cells for this study were CD45^+^CD3^–^CD56^+^. Immediately after sorting, we confirmed CXCR6 expression on sorted NK cells (**Supplemental Figure 1B**). Post-sort purity checks consistently demonstrated highly pure CXCR6^+^ and CXCR6^–^ NK cell populations *ex vivo* (**Figure 1A**).

We expanded CXCR6^+^ and CXCR6^–^ NK cells in the presence of IL-2 and IL-15 on irradiated and mitomycin C–treated RPMI-8866 feeder cells expressing CXCL16 mixed with irradiated allogeneic PBMC. This protocol expanded sorted CXCR6^+^ and CXCR6^–^ NK cell populations and generated sufficient cells for our subsequent phenotypic and functional analyses. After *in vitro* expansion, both cultures were negative for the presence of residual feeder cells (**Supplementary Figure 2**). Analysis of expanded NK cell cultures revealed that CXCR6^–^ NK cells had emerged in CXCR6^+^ NK cell cultures, which were now 63+/– 13% pure. In contrast, the purity of CXCR6^–^ NK cell cultures remained high (93+/–10%) (**Figure 1A**). CXCR6^–^ NK cell cultures were also consistently more viable. They expanded more readily than CXCR6^+^ NK cells despite the presence of CXCL16 on the surface of the feeder cell line RPMI-8866 (**Figure 1B** and **Supplementary Figure 1C**). We conclude that, while CXCR6^+^ and CXCR6^–^ NK cell populations expand, CXCR6^+^ PB-NK cells are less viable in our culture conditions.

The percent of CD56^bright^ NK cells and their CD56 mean fluorescence intensity (MFI) were significantly upregulated after *in vitro* expansion (**Figure 1C**), and both CXCR6^+^ and CXCR6^–^ PB-NK cells transitioned to a CD56^bright^ phenotype (**Figures 1C** and **Supplementary Figure 2**). These phenotypic changes are expected as IL-15 is known to upregulate CD56 (30).

To identify phenotypic differences between CXCR6^+^ and CXCR6^–^ PB-NK cells, we determined the *ex vivo* expression of NK cell differentiation, tissue residency markers, and effector function molecules using multiparameter flow cytometry. **Table 1** lists the individual markers, their functions, and our experimental rationale for their evaluations by flow cytometry or CyTOF. The gating strategy and representative flow plots for total, CXCR6^+^, and CXCR6^–^ PB-NK cells are shown in **Supplementary Figures 3A, B**. Our comparisons revealed that *ex vivo* (day 0), CXCR6^+^ and CXCR6^–^ PB-NK cells differ in their expression of several key NK cell markers associated with NK cell maturation and differentiation. Fewer CXCR6^+^ PB-NK cells expressed the activating receptors CD16 and NKG2C, the terminal differentiation marker CD57, and the Killer immunoglobulin-like receptor (KIR) KIR2DL1-DS1, when compared to CXCR6^–^ PB-NK cells (**Figure 1D**). However, the lectin-like activating receptor NKG2D and the natural cytotoxicity receptor NKp46 were expressed by a more significant percentage of CXCR6^+^, compared to CXCR6^–^ PB-NK cells. CD69, a marker frequently associated with early lymphocyte activation and/or tissue residency, and CD62L, a chemokine involved in lymph-node homing, are also preferentially expressed by CXCR6^+^ PB-NK cells (**Figure 1D** (19),). However, CXCR6^+^ PB-NK cells were less likely to express CX3CR1, which, like CD62L, is a chemokine receptor generally expressed by CD56^dim^ PB-NK cells (18).

**Table 1:** Analyzed cell surface and intracellular/nuclear markers, their scientific relevance, and references.

Marker	Functional/Phenotypic Relevance	References
CD56/NCAM	Phenotypic marker for identifying NK cells and subsets	(31)
CD16**/**FcγRIII	FcR mediating ADCC	(31, 32)
CXCR6	Chemokine receptor	(14, 26, 33)
T-bet	T-box transcription factor important for NK cell development/function	(12, 14, 17, 22)
Eomes	T-box transcription factor important for NK cell development/function, and tissue residence	(14, 17, 22, 34)
CD57	Terminal differentiation marker	(35–37)
KIR2DL1-2DS1	Inhibitory KIR (DL1)/Activating KIR (DS1) – important for licensing and regulating cytotoxicity	(38–40)
NKG2D	Activating receptor, binds MICA, MICB, UBLP1-6	(41–43)
NKG2A*	Inhibitory receptor, licensing and maturity status	(13, 39, 40, 44)
NKG2C	CMV-specific memory-like NK function	(45–51)
CD94	Forms heterodimers with NKG2A and/or NKG2C and binds HLA-E	(52)
NKp30*	Natural cytotoxicity receptor, constitutively expressed on NK cells	(53, 54)
NKp44*	Natural cytotoxicity receptor, expressed on activated NK cells	(53, 55, 56)
NKp46	Natural cytotoxicity receptor, constitutively expressed on NK cells	(53, 57)
CD137/4-1BB*	Activation-induced costimulatory molecule on NK cells	(58)
CD244/2B4*	Receptor with both activating and inhibitory roles	(59)
CD2*	Co-stimulatory receptor, particularly for ADCC	(60)
DNAM-1*	Activating receptor	(61)
CD69	Activation and/or tissue residency	(62)
CD107a	Present on lytic granules/lysosomes and is transported to the extracellular membrane upon degranulation.	(27)
Perforin	Generates pores in target cell membrane	(5, 63)
Granzyme B	A serine protease that cleaves intracellular caspases initiating apoptosis in target cells	(5, 63, 64)
IFN-γ	Pro-inflammatory cytokine	(65, 66)
TNF-α	Pro-inflammatory cytokine	(66)
CD49a*	Adhesion molecule marking tissue resident NK cells	(15, 67)
CD49e	Not expressed on liver resident NK cells	(68)
CX3CR1	Receptor for the chemokine CX3CL1 (fractalkine), adhesion/migration	(69)
CD62L	L-selectin, marks homing to lymph nodes	(70)
CCR9*	Chemokine receptor, homing to intestines	(71)
p-STAT5**	Signaling and transcription factor for cytokine receptors using the gamma chain	(72, 73)

*CyTOF analysis only; **flow cytometry analysis only.

### Phenotypic Differences Between CXCR6^+^ and CXCR6^–^ PB-NK Cells Are Stable After 14-Days of *In Vitro* Expansion

We used the same multi-parametric flow cytometry panels to phenotype day 14 NK cells. To be stringent, only CXCR6^–^ NK cells were analyzed within “CXCR6^–^ cultures”, and only CXCR6^+^ NK cells within “CXCR6^+^ NK cell cultures” (**Figure 1E**). Our gating strategy and representative flow plots are shown in **Supplementary Figure 2**. The phenotypic differences observed *ex vivo* between CXCR6^+^ and CXCR6^–^ NK cells remained robust after *in vitro* expansion.

Comparable to day 0, significantly fewer CXCR6^+^ NK cells expressed CD16, CD57, NKG2C, and KIR on day 14 (**Figures 1D, E** **)**. NKG2D expression increased in both the CXCR6^+^ and CXCR6^–^ subset, and, interestingly, CXCR6^+^ NK cells expressed a higher mean percentage of NKG2D. The difference in NKG2C expression between CXCR6^+^ and CXCR6^–^ NK cells was even more pronounced upon expansion, with NKG2C-expression mostly restricted to CXCR6^–^ NK cells (p<0.01). We encountered technical difficulties with our NKG2A antibody staining and were therefore unable to provide specific data on this inhibitory NK cell receptor by flow cytometry but have evaluated its expression by CyTOF as discussed later on in **Figure 7**. Nevertheless, on day 14, CD94, which forms heterodimers with NKG2A and NKG2C, was expressed by more CXCR6^+^ NK cells than CXCR6^–^ NK cells (CXCR6^–^, mean 69%; CXCR6^+^, mean 88%, p<0.05). While the mean expression for natural cytotoxicity receptor NKp46 was higher for CXCR6^+^ NK cells on day 14 of expansion, it did not differ significantly from CXCR6^–^ NK cells because of high donor variability.

The expression of the tissue-residency/activation marker CD69 also increased in both subsets. This expression pattern may be due to our stimulation conditions, as a prior study reported that IL-15 stimulated NK cells upregulate CD69 (74). However, CD69 was still more highly expressed on CXCR6^+^ NK cells (upon their stimulation) than CXCR6^–^ NK cells (**Figures 1D, E** **)**. Interestingly, CD62L expression had reversed by day 14 of culture. Fewer CXCR6^+^ NK cells expressed CD62L than CXCR6^–^ NK cells, reminiscent of liver resident NK cells (11, 14, 18, 26). Also, fewer CXCR6^+^ NK cells expressed CX3CR1 on day 14 of culture, although statistical significance was lost compared to our *ex vivo* analyses. We conclude from our data that phenotypic differences between CXCR6^+^ and CXCR6^–^ PB-NK cells are highly conserved on day 14 NK cells.

**Figure 2:**
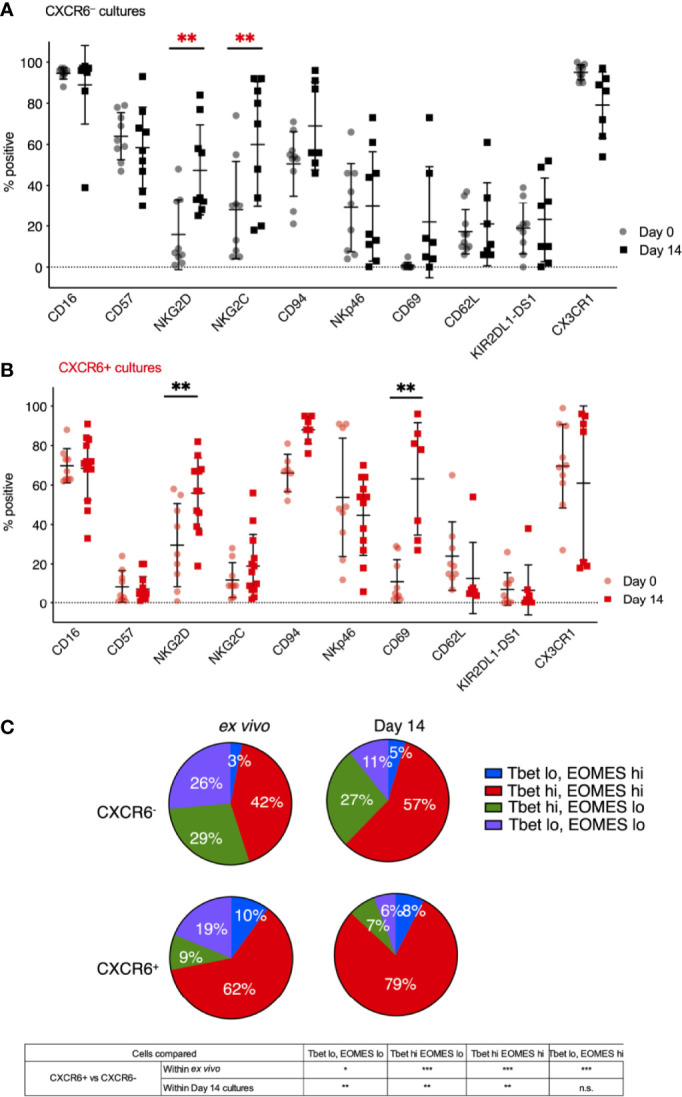
CXCR6^-^ NK **(A)** and CXCR6^+^ NK **(B)** remain phenotypically stable over 14 days in culture, except for increases in NKG2D, NKG2C, and CD69 (*n=7-9*). Not all cultures are paired for all markers, so a two-way ANOVA with Holm-Sidak multiple comparison’s tests was performed. P values are as denoted in the legend to **Figure 1**. **(C)** Four subsets of CXCR6^+^ and CXCR6^–^ PB-NK cells were identified in *ex vivo* and *in vitro* expanded NK cells based on their T-bet and Eomes expression: T-bet^lo^ Eomes^lo^ (purple), T-bet^lo^ Eomes^hi^ (blue), T-bet^hi^ Eomes^lo^ (green), and T-bet^hi^ Eomes^hi^ (red). Intranuclear transcription factor staining, and flow cytometry analyses was performed. Paired t-test was used for comparisons. Statistical significance is shown below. FMOs are shown in **Supplementary Figure 3C**. *p < 0.05; **p < 0.01; ***p < 0.001; ns, not significant.

We next performed a direct phenotypic comparison of freshly isolated (day 0) CXCR6^+^ (**Figure 2B**) and CXCR6^-^ PB-NK cells (**Figure 2A**) to their stimulated donor-matched counterparts to identify phenotypic changes in each subset that occurred during the *in vitro* expansion. We found that the phenotypes of CXCR6^+^ and CXCR6^–^ PB-NK cells were remarkably stable. NKG2D and NKG2C were exceptions: they were either upregulated on CXCR6^–^ PB-NK cells upon stimulation or NKG2D and NKG2C expressing CXCR6^-^ NK cells had expanded preferentially. NKG2C was consistently preferentially expressed on CXCR6^–^ rather than CXCR6^+^ NK cells. These NK cells closely resemble CMV-responsive memory-like NK cells (45–51, 75) and are CD57^hi^, NKG2C^hi^. NKG2D and CD69 significantly increased on CXCR6+ NK cells (p<0.01) (**Figure 2A**). We conclude that freshly isolated and expanded CXCR6^–^ PB-NK cells are similar to CMV-responsive memory-like NK cells in phenotype. In contrast, *ex vivo* and day 14 CXCR6^+^ NK cells displayed and retained a more immature phenotype characterized by lower CD16 expression, and a general lack of CD57 and KIR expression. This immature/immunotolerant phenotype, together with the expression of the tissue-residency marker CD69, bears a strong resemblance to CXCR6^+^ NK cells recently described in the human liver (16–19).

### CXCR6^+^ and CXCR6^–^ PB-NK Cells Express Distinct Combinations of T-Bet and Eomes

We and others previously published the T-bet and Eomes transcription factor profiles of liver and spleen NK cells (14, 16, 17, 19, 22, 34). In contrast to T-bet^hi^ Eomes^lo^ PB-NK cells, tissue-resident NK cells express Eomes at higher frequencies. Here, we clearly identified four subsets of CXCR6^+^ and CXCR6^–^ PB-NK cells based on their T-bet and Eomes expression: T-bet^lo^ Eomes^lo^, T-bet^lo^ Eomes^hi^, T-bet^hi^ Eomes^lo^, and T-bet^hi^ Eomes^hi^. To determine T-bet and Eomes expression for *ex vivo* and day 14 CXCR6^+^ NK cells, we performed intranuclear transcription factor staining and flow cytometry analyses (**Supplementary Figure 3C**). *Ex vivo*, 72% of CXCR6^+^ PB-NK cells expressed Eomes, while the majority (71%) of CXCR6^–^ PB-NK cells expressed T-bet (**Figure 2C**). T-bet^lo^ Eomes^hi^ and T-bet^hi^ Eomes^hi^ NK cells were significantly elevated in the CXCR6^+^ PB-NK cell subset compared to CXCR6^–^ PB-NK cells, which were mainly T-bet^hi^ Eomes^lo^ or T-bet^lo^ Eomes^lo^.

T-bet and Eomes expression were generally preserved throughout activation, despite previous data showing that prolonged stimulation with IL-15 induces Eomes expression in lymphocytes (72, 73). On day 14, 87% of CXCR6^+^ PB-NK cells expressed Eomes, while 62% of CXCR6^–^ PB-NK cells expressed Eomes (p < 0.005) (**Figure 2C**). NK cell activation and expansion decreased the frequency of double-negative T-bet^lo^ Eomes^lo^ NK cells in both CXCR6^+^ and CXCR6^–^ PB-NK cell subsets. These observed decreases correlated with increases in double-positive T-bet^hi^ Eomes^hi^ PB-NK cells. Both *ex vivo* and upon expansion, double-positive T-bet^hi^ Eomes^hi^ NK cells significantly dominated PB-NK cell cultures. They were more frequent in the CXCR6^+^ than in the CXCR6^–^ NK cell subset. On day 14, CXCR6^+^ T-bet^hi^ Eomes^lo^ PB-NK cells were rare (7%), while CXCR6^–^ T-bet^hi^ Eomes^lo^ NK cells comprised 27% of all CXCR6^–^ PB-NK cells (**Figure 2C**). We conclude that CXCR6^+^ PB-NK cells are more likely to express Eomes than their donor-matched CXCR6^–^ PB-NK cell counterparts.

### Day 14 CXCR6^+^ PB-NK Cells Phenotypically Resemble CXCR6^+^ NK Cells in the Human Liver and Spleen

**Figure 3:**
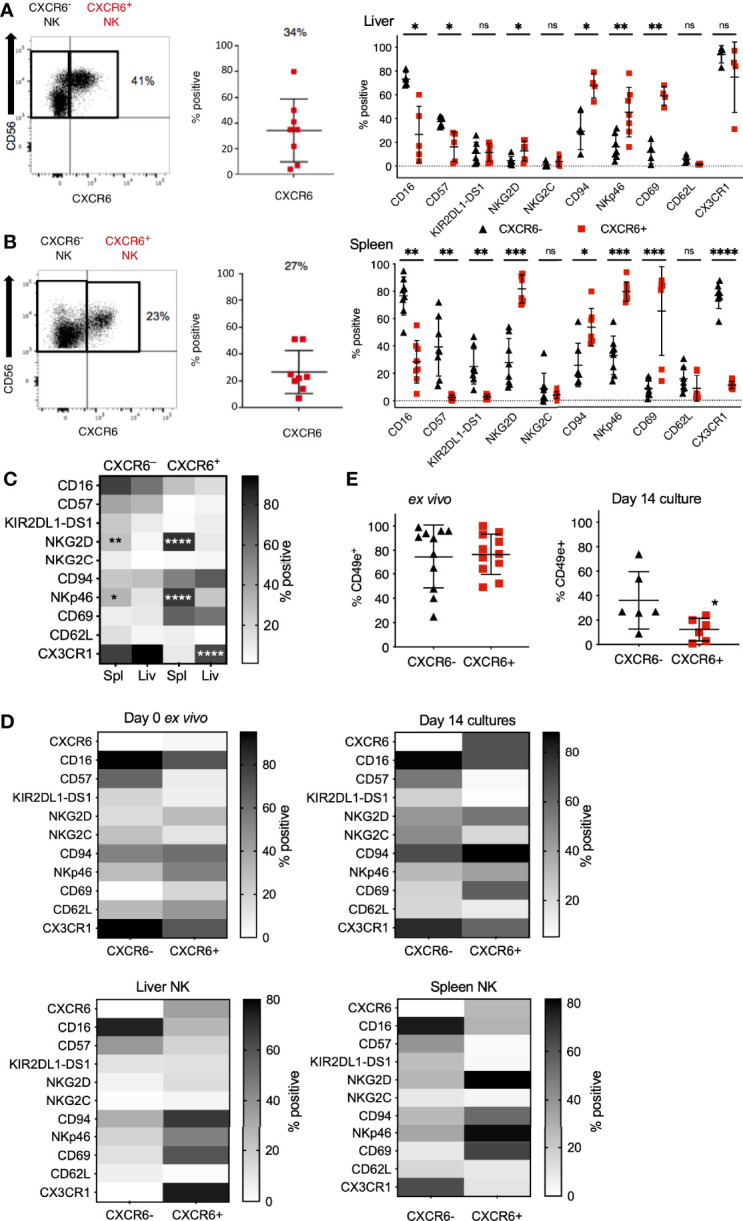
Phenotypic differences between CXCR6^-^ and CXCR6^+^ NK cells are augmented in the spleen compared to liver and PB-NK cells. CXCR6^-^ (black triangles) and CXCR6^+^ (red squares) liver and spleen NK cells were interrogated for various NK cell markers by flow cytometry. **(A)** CXCR6^+^ liver NK cells have a less mature, more tissue-resident phenotype than CXCR6^-^ liver NK (*n=5-7*). **(B)** Phenotypic differences between CXCR6- and + NK cell subsets are augmented in the spleen (*n=8*) (paired t-test for each marker). **(C)** Direct comparison of CXCR6^-^ spleen to CXCR6^-^ liver NK cells; and CXCR6^+^ spleen to CXCR6^+^ liver NK cells (two-way ANOVA with Sidak’s multiple comparison’s test). **(D)** Heat map summary comparing NK cell markers in CXCR6^-^ and CXCR6^+^ NK cells in *ex vivo* peripheral blood, 14-day cultures, liver, and spleen. Increasing intensity is equivalent to higher expression. Corresponding p values can be found in **Figures 1D, E**, **3A, B**. **(E)** CD49e is decreased on CXCR6^+^ NK cells compared to CXCR6^-^ NK after 14 days in culture. For *ex vivo* NK cells, *n=11*; for 14-day cultured NK cells, *n=6* (paired t-test). P values are as denoted in the legend to **Figure 1**. *p < 0.05; **p < 0.01; ***p < 0.001; ****p < 0.0001; ns, not significant.

We obtained liver perfusions from healthy cadaver donors to directly examine whether *ex vivo* and day 14 CXCR6^+^ PB-NK cells resemble CXCR6^+^ liver NK cells in phenotype and function using flow cytometry (**Supplementary Figure 4**). CXCR6 expression on human liver NK cells varied amongst donors in the liver perfusions we obtained, with a mean of 34% CXCR6^+^ liver-NK cells (**Figure 3A** and **Supplementary Table 1**) matching previous reports (16, 18).

As previously reported by us and others, CXCR6^+^ NK cells in the human liver were phenotypically immature (CD56^bright^, CD16^lo^, CD57^-/lo^), CD69^+^, and -T-bet^lo^, Eomes^hi^ (14, 16, 17, 22). Interestingly, freshly isolated and day 14 CXCR6^+^ PB-NK cells exhibit the same phenotype (CD56^bright^, CD16^lo^, CD57^lo^, CD69^+^) as human liver NK cells (**Figures 3A** and **2B**). Also, CXCR6^+^ liver NK cells and freshly isolated and day 14 CXCR6^+^ PB-NK cells were more likely to express CD94 and NKp46 than their CXCR6^–^ counterparts. We conclude that *ex vivo* and day 14 CXCR6^+^ PB-NK cells closely resemble CXCR6^+^ liver NK cells in phenotype.

Information concerning the phenotype and function of CXCR6^+^ and CXCR6^–^ NK cell subsets in the human spleen is limited. We previously published that NK cells from adult human liver and spleen tissue are phenotypically similar (14). However, we also found that human NK cells isolated from vaccinated humanized mouse liver, but not humanized mouse spleen, mediate immunological memory responses upon vaccination or viral infection (26). Therefore, we determined splenic CXCR6^+^ and CXCR6^–^ NK cell phenotypes and compared them to phenotypes of freshly isolated and day 14 PB-NK cells. We performed multi-parametric flow cytometry on single-primary-cell suspensions made from uninvolved spleen tissue from patients undergoing pancreatic ductal adenocarcinoma (PDAC) surgery, since spleen tissue from healthy donors was unavailable (see **Supplementary Figure 5** for gating strategy and representative flow plots). About 27% of human spleen-derived NK cells express CXCR6 **(** **Figure 3B** and **Supplementary Figure 5**), and splenic CXCR6^+^ NK cells closely resembled liver CXCR6^+^ NK cells in phenotype. Splenic CXCR6^+^ NK cells were predominantly CD56^bright^, CD16^lo^, CD57^lo^, KIR^lo^, NKG2C^lo^, high in their activation receptor expression (NKG2D, CD94, and NKp46), and expressed the tissue residency marker CD69. When compared to CXCR6^–^ NK cells, fewer CXCR6^+^ splenic NK cells express CD62L, albeit this difference was not statistically significant, and/or CX3CR1 (p<0.0001).

In **Figure 3C**, we statistically evaluated significant phenotypic differences in splenic and hepatic CXCR6^+^ and CXCR6^–^ NK cell subsets. Significant phenotypic differences include high numbers of NKG2D and NKp46 expressing splenic-NK cells (compared to liver NK cells) and a high frequency of CX3CR1^+^ NK cells in the splenic and hepatic CXCR6^–^ and hepatic CXCR6^+^ NK cell populations. In contrast, splenic CXCR6^+^ NK cells expressed CX3CR1 at low frequency, indicating differences in migratory capability between these tissue-resident NK cell populations (**Figure 3C**). The expression of CD16, CD57, KIR2DL1-DS1, NKG2C, CD69, and CD62L was similar on CXCR6^+^ and CXCR6^–^ NK cells in the spleen and the liver. For ease of comparison, we compiled our data into heat maps displaying the expression of all markers side by side for *ex vivo* and day 14 PB-NK cells, as well as spleen and liver NK cells (**Figure 3D**). Recently, the lack of CD49e (VLA-5 or fibronectin receptor) was designated as a liver-resident NK cell marker by us and others (14, 68), while the majority (about 80%) of PB-NK cells (CXCR6^+^ and CXCR6^–^) express CD49e *ex vivo* (**Figure 3E**). Hence, we investigated CD49e expression in our day 14 cultures and discovered that CD49e-expression is lost on both CXCR6^+^ and CXCR6^–^ NK cells after *in vitro* expansion, indicating that our culture conditions do not sustain CD49e expression on either subset, and there is a more significant decrease of CD49e expression on CXCR6^+^ NK cells (**Figure 3E**). The precise role of CD49e expression for NK cell development and tissue residency remains to be explored. We conclude that CXCR6^+^ and CXCR6^–^NK cells are distinct in phenotype regardless of tissue origin and that CXCR6^+^ PB-NK cells closely resemble hepatic and splenic CXCR6^+^ NK cells in phenotype, both *ex vivo* and on day 14 of expansion.

### Effector Functions of CXCR6^+^ PB-NK Cells Equal Those of CXCR6^–^ PB-NK Cells on Day 14

**Figure 4:**
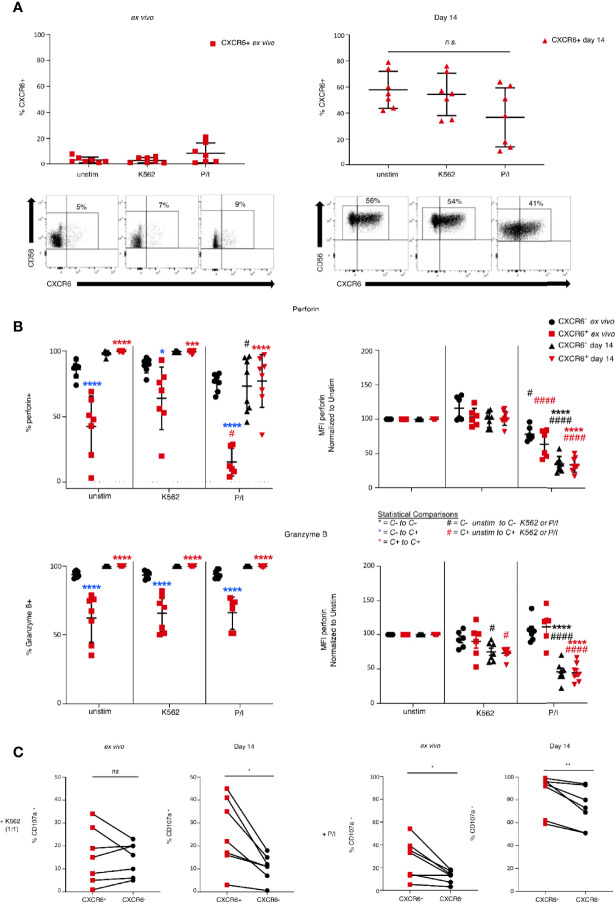
Differences in functional capability between *ex vivo* and 14 day-activated CXCR6- and + PB-NK cells. Day 0 (*ex vivo*) or day 14 cultured CXCR6- or + PB-NK cells were left unstimulated or stimulated with K562 (1:1) or PMA/ionomycin (P/I) for 4 hours. Multi-parametric flow cytometry was performed to determine changes in markers critical for NK cell function. **(A)** Changes in CXCR6 expression in *ex vivo* and day 14 cultured CXCR6^+^ NK cells after exposure to K562 or P/I. Representative flow plots are shown below the graphs. A one-way ANOVA with a Dunnett’s multiple comparison test was performed. **(B)** Changes in percent positive and MFI of perforin and granzyme B *Ex vivo* CXCR6^-^ NK (black circles); *ex vivo* CXCR6^+^ (red squares); day 14 CXCR6^-^ NK (black triangles); day 14 CXCR6^+^ NK, red triangles. Statistical comparisons are as designated in the key below. A two-way ANOVA with Tukey’s multiple comparison’s test was performed. One symbol, p < 0.05; two p < 0.01; three p < 0.001; four, p < 0.0001. **(C)** Percent CD107a^+^ (degranulating) NK cells in *ex vivo* and day 14-cultured CXCR6^+^ and CXCR6^-^ NK cells. CXCR6^+^ NK cells from 14-day cultures degranulate significantly more than CXCR6^–^ NK cells following stimulation with K562 (p < 0.05) or PMA/ionomycin (p < 0.01) (paired t-test) (*n=7*). ns, not significant.

To discern potential differences in effector function of CXCR6^+^ and CXCR6^–^ NK cells, we stimulated *ex vivo* and day 14 CXCR6^+^ and CXCR6^–^ PB-NK cells with either MHC class I-negative K562 target cells or phorbol 12-myristate 13-acetate (PMA) and the calcium ionophore ionomycin (P/I). Although not a physiologic stimulus, P/I stimulation can provide useful information on fundamental differences between CXCR6^+^ and CXCR6^-^ NK cell subset responses. Then we examined CD16, perforin, granzyme B, and CXCR6 expression, and levels of degranulation (CD107a). CXCR6 levels were unchanged on freshly isolated CXCR6^+^ PB-NK cells in response to K562 and P/I, whereas day 14 CXCR6^+^ PB-NK cells slightly down-regulate CXCR6 in response to P/I, but not to K562 target cell encounter (**Figure 4A**). Although this down-regulation is not statistically significant, it is consistent and becomes significant when using a paired t-test between matched samples (p<0.05).

NK cells kill target cells by perforin- and granzyme-induced apoptosis. Generally, mature NK cells have higher perforin and granzyme levels than immature NK cells (4). As expected, a high percentage of *ex vivo* analyzed phenotypically mature CXCR6^–^ PB-NK cells expressed high perforin and granzyme B levels compared to CXCR6^+^ PB-NK cells (**Figure 4B**). However, *in vitro* expansion sufficed for phenotypically immature CXCR6^+^ NK cells to express perforin and granzyme B levels similar to that of CXCR6^–^ NK cells (**Figure 4B**). Stimulation of CXCR6^+^ and CXCR6^–^ NK cell cultures with K562 target cells did not significantly change levels of perforin and granzymes. However, upon stimulation with P/I, the frequency of perforin expressing CXCR6^+^ and CXCR6^–^ NK cells decreased in *ex vivo* and day 14 populations, likely due to enhanced degranulation. We also analyzed changes in the Mean Fluorescence Intensity (MFI) of perforin expression following stimulation by normalizing unstimulated cells to 100% and measuring the percent change in perforin MFI as a further measure of cytotoxicity. K562 stimulation did not significantly change perforin MFI for any NK cell subsets (**Figure 4B**). Both *ex vivo* and day 14 CXCR6^+^ and CXCR6^–^ NK cells decrease perforin MFIs, but *ex vivo* NK cells did so to a lesser extent than day 14 NK cells. In other words, *in vitro* expanded and restimulated NK cells are releasing more perforin into target cells than *ex vivo* NK cells upon P/I stimulation. The MFI of granzyme B expressing NK cells follows a similar pattern as perforin (**Figure 4B**).

CD107a, or Lysosome-Associated Membrane Protein-1 (LAMP-1), is an indicator of NK cell effector function and can only be detected if degranulation of cytotoxic granules is occurring (27, 28, 76). We next determined differences in the degranulation capacity of CXCR6^+^ and CXCR6^–^ PB-NK cells, again using *ex vivo* and day 14 activated NK cells and K562 or P/I stimulation. We did not observe a difference in CD107a in *ex vivo* CXCR6^+^ and CXCR6^–^ PB-NK cells in response to K562. However, *ex vivo* CXCR6^+^ PB-NK cells degranulate more than CXCR6- PB-NK cells in response to P/I (p<0.05) (**Figure 4C**). CXCR6^+^ PB-NK cells, despite their “immature” phenotype, also degranulate significantly more than CXCR6^–^ NK after *in vitro* expansion when stimulated with either K562 targets or P/I *in vitro* (**Figure 4C**). Our data demonstrate that CXCR6^+^ PB-NK cells resemble day 14-activated CXCR6^–^ PB-NK functionally in terms of perforin and granzyme B expression and degranulate more than CXCR6^–^ NK cells in response to K562 and P/I on day 14. Degranulation is robust in this subset despite being widely considered an immature NK cell subset when examined *ex vivo* (16, 19). Hence, CXCR6^+^ PB-NK cells show functional plasticity in that they are less potent cytotoxic effectors immediately *ex vivo*, but with appropriate stimulation their ability to perform cytotoxic effector functions becomes greatly enhanced despite their immature phenotype, making them functionally equal to CXCR6^–^ PB-NK cells.

### *Ex Vivo* and Day 14 CXCR6^+^ and CXCR6^–^ PB-NK Cells Produce Similar Amounts of IFNγ and TNFα

To discern the functional plasticity and potential differences in effector function of CXCR6^+^ and CXCR6^–^ NK cells, we stimulated *ex vivo* and day 14-activated CXCR6^+^ and CXCR6^–^ PB-NK cells with K562 or P/I and determined their cytokine production by intracellular cytokine staining and multiparameter flow cytometry. CXCR6^+^ and CXCR6^–^ PB-NK cells produce similar amounts of IFNγ and TNFα in response to both K562 or P/I stimulation in terms of frequency of parent and MFI (**Supplementary Figure 6**).

### Liver and Spleen NK Cells Exhibit Reduced Cytotoxic Activity and Respond Poorly to IL-2 Stimulation Compared to PB-NK Cells

**Figure 5:**
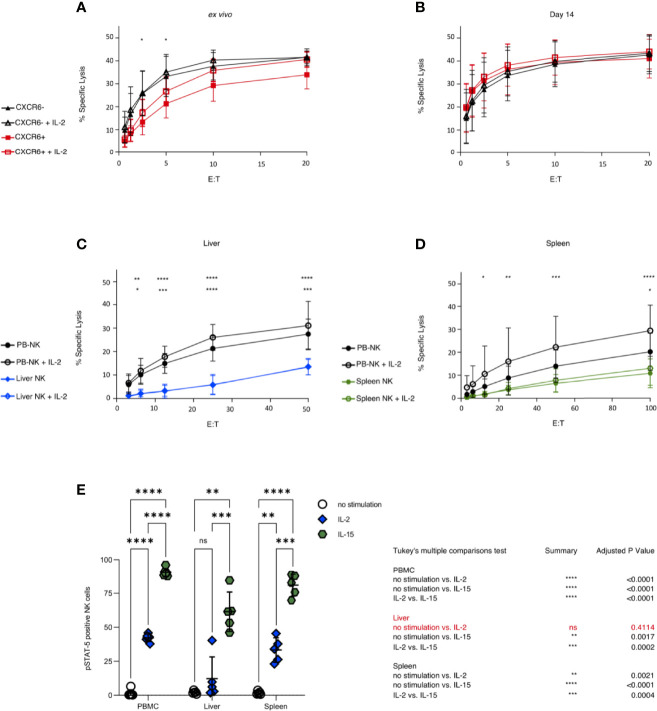
PB-NK cells show higher cytotoxicity against K562 than liver and spleen NK cells, and CXCR6^+^ PB-NK cells are functionally equivalent to CXCR6^-^ PB-NK cells after 14 days in culture. Sorted CXCR6^-^ and + NK cells were used as effectors starting at a 20:1 effector to target ratio (E:T). Percent specific lysis is shown as a function of E:T. All assays were run in duplicate or triplicate. **(A)** Freshly sorted CXCR6^-^ NK cells possess a higher cytotoxic function against K562 than CXCR6^+^ NK at lower E:T *ex vivo* (*n=5*). **(B)** CXCR6^-^ and CXCR6^+^ NK cells are virtually identical in their ability to kill K562 targets by 14 days post-activation in culture (*n=10*). **(C)** Unsorted *ex vivo* bulk liver lymphocytes have low cytotoxic capability against K562 and are unresponsive to IL-2 (*n=5*). Mean of 42% NK cells in bulk liver cells (range 22-61%) **(D)** Bulk spleen lymphocytes also exhibit low cytotoxicity against K562 but are slightly responsive to IL-2 at higher E:T ratios (*n=8*). Mean of 11.4% NK cells in bulk spleen cells (range 7-14%). Two-way ANOVA with Tukey’s multiple comparison test was used to compare cytotoxic effector function. P values are as described in **Figure 1**. The top row of p values designates significance between CXCR6- and + **(A, B)** or between PB and liver or spleen **(C, D)**. The bottom row of p values is within each tissue plus or minus IL-2. For complete statistical analysis, see **Supplementary Table 6**. **(E)** Liver NK cells (CD45^+^, CD56^+^, CD3^–^) are virtually unresponsive to IL-2 stimulation as measured by STAT5 phosphorylation. Phosphorylation of STAT5 in bulk NK cells in PB, liver, and spleen following stimulation with IL-2 (10 ng/mL) (blue diamonds) or IL-15 (50 ng/mL) (green hexagons) (*n=5*). Tukey’s multiple comparisons test was used to determine significance between no stimulation (open circles) vs IL-2 or IL-15 treatment, or IL-2 vs IL-15. p < 0.05 was considered significant. (*p < 0.05; **p < 0.01; ***p < 0.001; ****p < 0.0001; ns, not significant).

We further explored potential functional differences between CXCR6^+^ and CXCR6^–^ PB-NK cell subsets by examining their killing of K562 targets *via* chromium release assay. We used either freshly sorted CXCR6^+^ and CXCR6^–^ PB-NK cells or day 14 CXCR6^+^ and CXCR6^–^ PB-NK cell cultures as effector cells. Freshly isolated CXCR6^–^ PB-NK cells were maximally induced, and the addition of IL-2 did not markedly improve their cytotoxic function. In contrast, freshly isolated CXCR6^+^ PB-NK cells were not as robust in their ability to lyse K562 targets as their CXCR6^–^ NK cell counterparts, especially at lower E:T ratios (p<0.05) (**Figure 5A**). Interestingly, freshly isolated CXCR6^+^ PB-NK cells could be induced to match CXCR6^–^ PB-NK cell baseline killing by IL-2 stimulation during the killing assay, especially at higher E:T ratios (**Figure 5A**). Hence, although CXCR6^+^ PB-NK cells exhibit lower cytotoxic function *ex vivo*, they are more responsive to IL-2 and match CXCR6^–^ PB-NK cell cytotoxic capacity in the presence of this cytokine. Remarkably, CXCR6^+^ and CXCR6^–^ PB-NK cells were equivalent in lysing K562 target cells on day 14 of expansion and killing was more effective at lower E:T ratios (**Figure 5B**).

CXCR6^+^ NK cells are abundant in the liver and spleen [**Supplementary Table 1** and (16, 19)]. Unfortunately, we did not obtain enough liver perfusate or splenic tissue to sort CXCR6^+^ and CXCR6^–^ NK cells subsets for direct functional comparisons. Thus, we compared the ability of bulk liver (mean 42.4% NK cells) and spleen (mean 11.4% NK cells) cells to lyse K562 target cells. Liver NK cells were less robust in their ability to kill MHC class I-negative targets than PB-NK cells, presumably due to their lack of KIR expression and incomplete licensing (77). Indeed, the ability of liver NK cells to lyse K562 targets was significantly reduced, and liver NK cells were unresponsive to IL-2 stimulation compared to PB-NK controls (**Figure 5C**). Spleen NK cells also displayed very low cytotoxicity against K562 targets; they were minimally responsive to IL-2 and only at higher E:T ratios (**Figure 5D**). *Ex vivo* CXCR6^-^ PB-NK cells were very efficient cytotoxic effectors against K562 targets, as were freshly sorted and day 14 CXCR6^+^ and CXCR6^–^ PB-NK cells. We conclude that liver and spleen NK cells exhibit reduced cytotoxic activity and respond poorly to IL-2 stimulation compared to freshly sorted and day 14 CXCR6^+^ and CXCR6^–^ PB-NK cells.

Given the poor responsiveness to IL-2, we asked whether STAT-5 is phosphorylated upon IL-2 and/or IL-15 stimulation of bulk liver, spleen, and PBMC-NK cells. In agreement with our cytotoxicity assays (**Figures 5A–D**), bulk PBMC-NK and spleen NK cells phosphorylated STAT-5 upon stimulation with IL-2 or IL-15. In contrast, bulk liver NK cells did not respond to IL-2 stimulation but responded vigorously to IL-15 stimulation (**Figure 5E**). Our data reveal a surprising difference between liver and spleen NK cells. Liver NK cells cannot phosphorylate STAT-5 upon IL-2 stimulation. However, this defect is specific to IL-2 signaling as it is not observed when liver NK cells are stimulated with IL-15.

### Day 14 CXCR6^+^ PB-NK Cells Possess Superior ADCC Function, While Bulk Spleen and Liver NK Cells Significantly Differ in Their Cytotoxic and ADCC Capabilities

**Figure 6:**
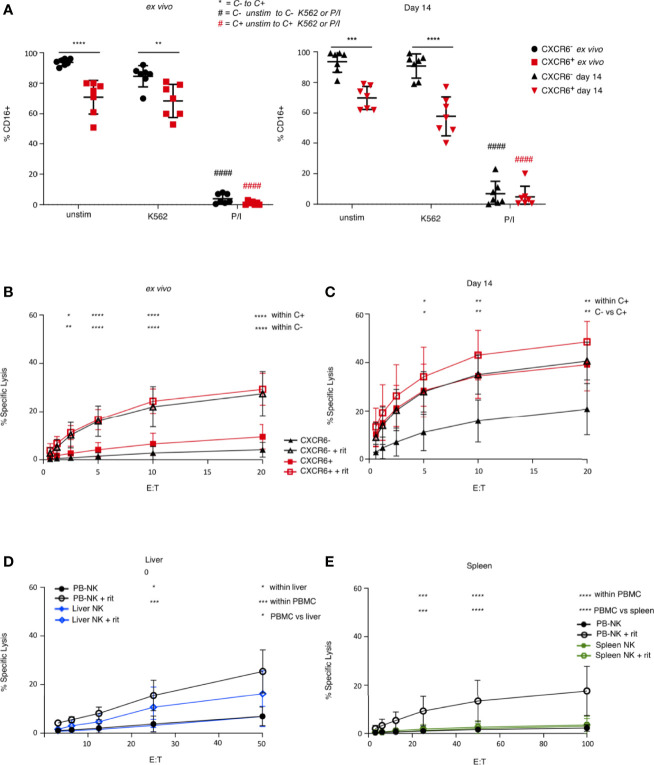
Comparison of ADCC function in *ex vivo*, day 14-cultured, liver, and spleen NK cells. **(A)** CXCR6^+^ NK cells express less CD16 than CXCR6^-^ NK cells, and down-regulate CD16 in response to K562 target cells after 14 days in culture, although not significantly (*n=7*). A two-way ANOVA with Tukey’s multiple comparisons test was performed. **(B)** *Ex vivo* CXCR6- and + NK cells have equal capacity to perform ADCC (*n=5*). Freshly sorted or 14 day-cultured CXCR6^+^ and CXCR6^–^PB-NK cells or bulk liver or spleen cells were incubated with Raji targets with or without rituximab (rit). NK cell percentages in bulk liver and spleen cells were as described in the legend to **Figure 5**. Assays were performed in duplicate or triplicate. **(C)** 14 day-cultured CXCR6^+^ NK cells exhibit enhanced ADCC function compared to CXCR6^-^ NK cells (*n=5*). **(D)** Unsorted liver and PB-NK cells have comparable ADCC activity (*n=5*). **(E)** Unsorted spleen NK cells have decreased ADCC compared to PB and liver NK cells (*n=8*). A two-way ANOVA with Tukey’s multiple comparisons test was performed. p values for statistically significant relationships are shown. For complete statistical analysis, see **Supplementary Table 8**. (*p < 0.05, **p < 0.01, ***p < 0.001, ****p < 0.0001).

We next investigated whether CD16 is down-regulated differentially on CXCR6^+^ and CXCR6^–^ PB-NK cells after stimulation. We found that CD16 was not significantly downregulated in response to K562 on either PB-NK cell subset when stimulated *ex vivo.* However, CD16 expression was downregulated in response to P/I on both subsets. Interestingly, day 14 CXCR6^+^ and CXCR6^–^ PB-NK cells down-regulated CD16 in response to P/I. Only expanded CXCR6^+^ PB-NK cells down-regulated CD16 in response to K562, albeit the observed decrease in CD16 expression is not statistically significant (**Figure 6A**).

We next determined the ADCC capability of freshly sorted *ex vivo* and day 14-activated PB-NK cells. Surprisingly, *ex vivo*, CXCR6^+^, and CXCR6^–^ PB-NK cells were equal in their ability to lyse Raji targets in the presence of rituximab despite CXCR6^+^ PB-NK cells being CD16^lo^ (**Figure 6B**). Even more surprising was that day 14 CXCR6^+^ PB-NK cells were significantly better ADCC effectors than their CXCR6^–^ counterparts (**Figure 6C**). Interestingly, day 14 CXCR6^+^ PB-NK cells killed Raji cells without rituximab at levels equivalent to rituximab-dependent killing observed with day 14 CXCR6^–^ NK cells. We conclude that CXCR6^+^ PB-NK cells functionally mature upon activation to develop superior ADCC, surpassing highly activated CXCR6^–^ PB-NK cells.

We next compared ADCC functions of bulk liver (mean 42.4% NK cells) and spleen (mean 11.4% NK cells) NK cells to bulk PB-NK control cells. Surprisingly, we found that bulk liver NK cells were significantly better mediators of ADCC than bulk spleen NK cells and similar to PB-NK cells in their ability to perform ADCC. In contrast, spleen NK cells were virtually unable to perform ADCC (**Figures 6D, E**). We previously demonstrated that liver NK cells are relatively poor mediators of cytotoxicity (when stimulated with K562 target cells) and are entirely unresponsive to IL-2 stimulation (**Figures 5C–E**). We conclude that liver and spleen NK cells are phenotypically similar and contain a similar percentage of CXCR6^+^ NK cells but differ in their cytotoxic and ADCC capabilities.

### Day 14 CXCR6^+^ and CXCR6^–^ PB-NK Cells Are Comprised of Multiple Subsets

**Figure 7:**
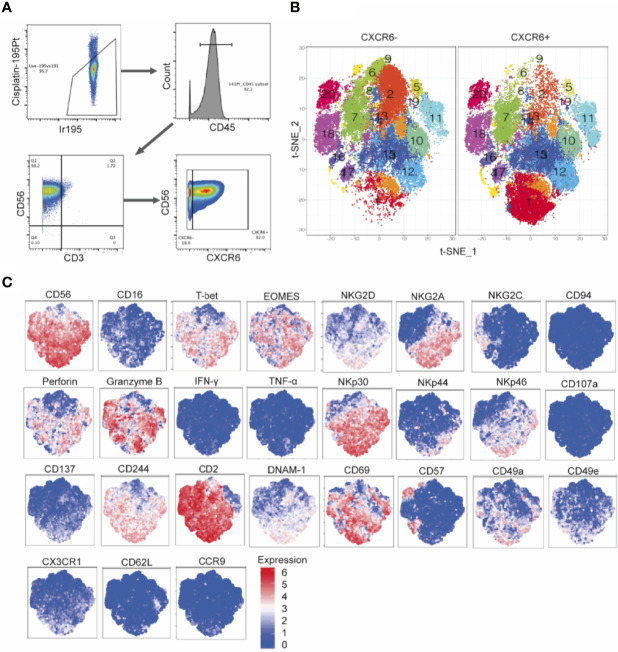
Mass cytometry immunophenotyping detects subsets of CXCR6^+^ NK cells with diverse expression patterns, distinct from that of CXCR6- NK cells. **(A)** Hierarchical gating strategy of cultured PBMC derived CXCR6^+^ and CXCR6^-^ NK cell cultures. CXCR6^+^ and CXCR6^-^ NK cells were single cell sorted from PBMCs (*n=8* donors) and cultured with IL-15 and CXCL16 feeder cells for 2-7 weeks. Cultured cells were stained for 32 cell surface and intracellular targets and analyzed by mass cytometry. **(B)** t-Distributed Stochastic Neighbor Embedding (t-SNE) dimensionality reduction and Rphenograph clustering of CXCR6^+^ and CXCR6^-^ NK cells. **(C)** Expression patterns of selected markers.

To identify phenotypic and potentially functional subsets in CXCR6^+^ and CXCR6^–^ PB-NK cell populations, we sorted and expanded CXCR6^+^ and CXCR6^–^ PB-NK cells for about seven weeks using the same protocol as for flow cytometry-based phenotypic and functional analyses. This approach generated several million NK cells of each subset for analysis by CyTOF (**Figures 7A–C**). **Table 1** lists the individual markers, their functions, and our experimental rationale for their evaluations. Rphenograph clustering of our CyTOF data revealed that both CXCR6^+^ and CXCR6^–^ PB-NK cell populations comprised 20 clusters (**Figures 7A–C**). Most clusters were similarly frequent between CXCR6^+^ and CXCR6^–^ PB-NK cell populations. However, cluster 1 was expanded in the CXCR6^+^ vs. the CXCR6^–^ PB-NK cell population. Similar to our flow cytometry data, EOMES was expressed by CXCR6^+^ PB-NK cells, and both CXCR6^+^ and CXCR6^–^ PB-NK cell populations were comprised of T-bet^lo^ Eomes^lo^, T-bet^lo^ Eomes^hi^, T-bet^hi^ Eomes^lo^, and T-bet^hi^ Eomes^hi^ NK cells, albeit at different ratios. In agreement with our flow cytometry data, 23% of expanded CXCR6^+^ PB-NK cells were CD56^hi^ and expressed CD69, NKG2D, NKG2A, Perforin, Granzyme B, T-bet, and Eomes (cluster 1) (**Figure 7B**). They also expressed high levels of NKp30, CD2, NKp46, CD244, and DNAM-1, intermediate levels of CD49a, CX3CR1, and CD137, and low levels of NKG2C, CD57, CD62L, CD49e, and CD16. Two additional clusters (12 and 15) followed a similar trend but expressed less DNAM-1 and NKG2D than cluster 1 NK cells (**Figure 7B**). A recent study demonstrated that the transcription factor PLZF is expressed by intrahepatic CD56^hi^, CXCR6^+^, CD69^+^ NK cells, but not by freshly isolated CXCR6+ PB-NK cells (11). As we performed our studies before the publication of these data, we did not evaluate PLZF expression levels in our samples.

In contrast, the expanded CXCR6^–^ PB-NK cell population did not contain many “cluster 1” NK cells (< 3%) but were enriched in clusters 2, 5, 6, 8, and 9, and to a lesser extent, cluster 18 NK cells (**Figure 7**). Despite their expansion in the presence of IL-15, CXCR6^–^ PB-NK cells expressed T-bet and were CD56^lo^, Eomes^lo^, and CD69^lo^. Expanded CXCR6^–^ PB-NK cells did not express much NKG2A but expressed perforin and granzyme B at varying levels depending on the cluster. We conclude that day 14 CXCR6^+^ and CXCR6^–^ PB-NK cells are comprised of multiple subsets based on their cell surface receptor, effector function molecule, and transcription factor expression. Our data uncover a previously underappreciated phenotypic and functional plasticity in both NK cell subsets.

## Discussion

Our study resulted in several interesting discoveries. In agreement with other reports (11, 13, 14, 16–18), we found that CXCR6^+^ PB-NK cells phenotypically resemble CXCR6^+^ NK cells found in human liver and spleen (CD69^+^, CD57^lo^, NKG2C^lo^, and NKG2A^+^). In contrast, CXCR6^–^ PB-NK cells resembled a subset of CD57^hi^, NKG2C^hi^, NKG2A^–^ NK cells that preferentially expand following CMV infection or reactivation (37, 51, 78, 79). CXCR6^+^ and CXCR6^–^ PB-NK cells differ in their T-bet and Eomes expression profiles. Eomes expression was more frequent in CXCR6^+^ PB-NK cells, which also contained a small percentage of Eomes^+^, T-bet^lo^ NK cells not found in the CXCR6^–^ PB-NK cell subset. Both CD56^hi^ and CD56^lo^ PB-NK cells expressed CXCR6 *ex vivo*. NK cells can upregulate the expression of CD56 upon activation, and CD56 expression has previously been used as a marker of NK cell activation (80). Upon *in vitro* expansion in the presence of IL-2 and IL-15, NK cells became predominantly CD56^hi^ and retained their immature phenotype but became strongly cytotoxic.

The finding that CXCR6^+^ PB-NK cells are phenotypically similar to CXCR6^+^ NK cells from the liver and spleen raises the question of where CXCR6^+^ PB-NK cells originate. CXCL16, the ligand for CXCR6, is expressed by hepatic sinusoidal endothelial cells in mice and humans and retains CXCR6^+^ NK cells in the liver (18, 25). CXCL16 also plays an essential role in providing survival signals for CXCR6^+^ lymphocytes (18, 25). A liver transplant study found that Eomes^hi^ liver NK cells remained in the transplanted liver for at least 13 years while Eomes^lo^ NK cells were seen migrating from the liver to recirculate (34). NK cells also undergo differentiation in other secondary lymphoid organs throughout the body (81). Therefore, CXCR6^+^ PB-NK cells may derive from secondary lymphoid tissues or from circulating peripheral CXCR6^+^, Eomes^lo^ NK cells recruited to the liver by CXCL16. Once in the liver, Eomes^lo^ NK cells may upregulate Eomes expression, perhaps upon exposure to IL-12, IL-15, and/or TGF-β (34, 73, 82). Alternatively, subsets of CXCR6^+^ NK cells may develop *in situ*; circulating CXCR6^+^, Eomes^lo^ NK cells and liver resident Eomes^hi^ NK cells may be distinct NK cell subsets. Whether splenic CXCR6^+^ NK cells circulate or remain in the spleen remains to be experimentally determined.

In contrast to the human spleen, where CXCR6^+^ NK cells are abundant, CXCR6^+^ NK cells are rarely found in the mouse spleen (24, 25, 83). However, CXCL16 expression has been reported in mice on splenic CD8^+^, CD11c^+^ dendritic cells and is induced further by various microbial stimuli (84). T cell areas of the murine splenic white pulp and splenic red pulp can also express CXCL16, as do cells lining vascular sinuses (84). Hence, constitutively expressed or inducible CXCL16 may support the retention of CXCR6^+^ immune cells, including small numbers of NK cells, in the spleen and the liver of mice and humans.

We have already published that, overall, spleen NK cells are more mature in phenotype and are more likely to contain higher proportions of T-bet^+^ NK cells (14). Our data demonstrate specialized effector functions of splenic and hepatic NK cells, both of which have substantial percentages of CXCR6^+^ NK. However, bulk spleen and liver NK cells were both unable to kill K562 target cells. Interestingly, bulk liver NK cells were unresponsive to IL-2 stimulation and did not phosphorylate Stat5 in response to IL-2. In contrast, spleen NK cells were more capable of induction by IL-2 in cytotoxicity assays against K562 cells (at higher effector to target ratios) and phosphorylated Stat5 at a frequency similar to bulk PBMC-NK cells. Interestingly, bulk PBMC, liver, and spleen NK cells responded to IL-15 stimulation equally well and phosphorylated Stat5. Thus, the lack of Stat5 phosphorylation and activation observed in liver NK cells is specific to IL-2 stimulation rather than a cell-intrinsic defect. Stat5 binding sites have been identified in genes modulating NK cell development and function, including Eomes, T-bet, perforin, granzymes, and IFN-γ. Additionally, Stat5 activation augments NK cell survival (85, 86). Also, in IL-2 stimulated lymphocytes, Stat5 phosphorylation is required for cell cycle progression (72, 87). However, we did not have enough sorted cells to examine Ki67 expression in our experiments. In the future, it would be interesting to determine the proliferative capacity of liver and spleen NK cells upon IL-2 and/or IL-15 stimulation and any differences in cytokine receptor expression by NK cells in each respective tissue.

Over the past decade, we and others discovered antigen-specific immunological-memory-capable NK cell subsets in multiple species (24–26, 83, 88, 89). In mice, lung and liver resident CXCR6^+^ NK cells mediate adaptive immunity to viral antigens and inactivated viruses, while CXCR6^–^ NK cells from the same animals do not. Also, CXCR6 regulates hepatic but not splenic-NK cell homeostasis (25). Similar to our data in mice, human CXCR6^+^ liver NK cells also mediated antigen-specific memory responses to viral antigens in humanized mice, while splenic NK cells did not (26). Notably, rhesus macaques possess long-lived antigen-specific NK cell memory for up to five years following HIV vaccination and infection. These memory NK cells originated from both the liver and spleen. However, their CXCR6 status was not determined (90). In contrast to mice, humanized mice and humans harbor many CXCR6^+^ splenic NK cells. In humans, splenic CXCR6^+^ NK cells closely resemble CXCR6^+^ liver resident NK cells in phenotype. However, their cytotoxicity and ADCC killing functions are poor. These data may help explain why splenic NK cells in mice and humanized mice do not mediate adaptive immunity, while both splenic and hepatic NK cells have been suggested to do so in non-human primates. Additional work is needed to determine whether CXCR6/CXCL16 interactions regulate splenic and hepatic human NK cells’ survival and memory capability.

Phenotypic differences between CXCR6^+^ and CXCR6^–^ PB-NK cells observed *ex vivo* largely remained after 14-days of *in vitro* expansion. However, CXCR6^–^ PB-NK cells expanded better than CXCR6^+^ PB-NK cells despite CXCL16-expression on 8866 feeder cells, aiding immune cell survival by inducing anti-apoptotic molecules (25, 91). As it is not known if CXCL16 splice variants differ in their signaling or biological effects, we expressed both variants on 8866 feeder cells used to stimulate PB-NK cells. CXCR6^+^ PB-NK cells, expanded with CXCL16-expressing 8866 cells, IL-2, and IL-15, closely resembled CXCR6^+^ liver and spleen NK cells. As previously reported, IL-15 induced the expression of Eomes (34), albeit more so in CXCR6^+^ than CXCR6^–^ PB-NK cells. Interestingly, the long-term expansion of CXCR6^–^ PB-NK cells on CXCL16-expressing 8866 feeder cells did not result in CXCR6-expression on previously CXCR6 negative cells. In contrast, some CXCR6^–^ NK cells emerged in the CXCR6^+^ PB-NK cell culture. CXCR6^–^ PB-NK cells expanded better than CXCR6^+^ PB-NK cells (**Figure 1**). We speculate that emerging CXCR6^–^ NK cells are the offspring of a very few CXCR6^–^ PB-NK contained within the original sort, outcompeting CXCR6^+^ PB-NK cells during expansion. However, we cannot formally exclude the possibility that CXCR6^+^ PB-NK cells give rise to CXCR6^–^ PB-NK cells during *in vitro* culture.

NK cells in the CXCR6^+^ NK cell subset markedly increased CD69 expression compared to CXCR6^–^ NK cells upon *in vitro* stimulation. We found this difference surprising as both subsets were activated under identical conditions. Whether this difference is due to a preferential outgrowth of CD69-expressing CXCR6^+^ NK cells or due to CD69-upregulation remains to be determined. Both *ex vivo* and day 14 CXCR6^+^ PB-NK cells exhibited a phenotype closely resembling their liver and secondary lymphoid tissue counterparts [**Figure 3D** and (16–19)]. Also, despite their reduced expansive capacity and differential phenotype, CXCR6^+^ PB-NK cells became functionally equivalent to CXCR6^–^ PB-NK after 14-days of culture. IFNγ and TNFα production were stimulus-dependent and similar between CXCR6^+^ and CXCR6^–^ PB-NK cells, *ex vivo* and after *in vitro* expansion. CXCR6^+^ PB-NK cells remained “phenotypically immature” but upregulated perforin and granzyme B proteins during the two-week *in vitro* culture and killed K562 target cells as well as “phenotypically mature” day 14 CXCR6^–^ NK cells.

Many potential NK cell subsets have been identified in human bulk PB-NK cells (92), albeit their particular biological importance remains to be fully understood. Using CyTOF analysis of long-term CXCR6^+^ and CXCR6^–^ PB-NK *in vitro* cultures, we found that expanded CXCR6^+^ and CXCR6^–^ PB-NK cells are comprised of multiple (20) subsets based on their cell surface receptor, effector function molecule, and transcription factor expression. Our results demonstrate a previously unappreciated phenotypic and functional plasticity. CXCR6^+^ and CXCR6^–^ PB-NK cells are neither homogenous NK cell populations nor terminally differentiated but are comprised of subsets with potentially specialized homing capacities and functions. Interestingly, all but one PB-NK cell subset expressed high levels of CD2, a glycoprotein and ligand for LFA3. CD2 augments NK cell cytotoxicity as a co-stimulator and by recruiting CD16 into the immunological synapse and can be upregulated on lymphocytes upon prolonged stimulation with IL-2 (60).

Surprisingly, bulk spleen and liver NK cells differed significantly in their ADCC capabilities. Splenic NK cells were unable to mediate ADCC, while liver NK cells mediated strong ADCC. What causes this dramatic difference in ADCC ability is currently unknown. Activation-induced loss of CD16 from the NK cell surface is well-established and due to cleavage by metalloproteases ADAM 10 and 17 (93, 94). We also observed a decrease in CD16 expression on day 14 CXCR6^+^ and CXCR6^–^ PB-NK cells following stimulation with P/I. While loss of CD16 from the cell surface is thought to inhibit ADCC function, the up-regulation of NKG2D in day 14 CXCR6^+^ NK cells may enhance ADCC function in this subset (95). Overall, day 14 CXCR6^+^ NK cells had superior ADCC function compared to CXCR6^–^ PB-NK cells. Therefore, it should not be assumed that CXCR6^+^ NK cells are functionally immature based on their phenotype.

Our data inform on the level of plasticity and unique functionality of CXCR6^+^ and CXCR6^–^ NK cell subsets present in human PB, liver, and spleen. Cytokine-activated or chimeric-antigen-receptor expressing day 14 NK cell infusion products are increasingly used to treat human diseases (96). Our findings may allow for the selection of superior NK cell subsets for infusion products increasingly used to treat human diseases.

## Data Availability Statement

The raw data supporting the conclusions of this article will be made available by the authors, without undue reservation.

## Ethics Statement

The studies involving human participants were reviewed and approved by Institutional Review Board at Baylor College of Medicine. The patients/participants provided their written informed consent to participate in this study.

## Author Contributions

Conceptualization was performed by SP. Formal analysis was performed by LA, GH, SA, LB, AV-H, and SP. Funding acquisition was performed by SP. Investigation was performed by LA, GH, and SA. Methodology was designed by LA, GH, SA, AV-H, and SP. Project administration and supervision was performed by SP. Validation was performed by LA, GH, SA, and SP. Visualization was performed by LA, SA, and SP. Writing was performed by LA, SA, and SP. All authors contributed to the article and approved the submitted version.

## Funding

This work was supported by NIH grants RO1 AI116282 and R01 AI161014 to SP, and by seed funds to SP from Baylor College of Medicine and The Scripps Research Institute (to SP).

## Conflict of Interest

The authors declare that the research was conducted in the absence of any commercial or financial relationships that could be construed as a potential conflict of interest.

## Publisher’s Note

All claims expressed in this article are solely those of the authors and do not necessarily represent those of their affiliated organizations, or those of the publisher, the editors and the reviewers. Any product that may be evaluated in this article, or claim that may be made by its manufacturer, is not guaranteed or endorsed by the publisher.
