# Supplementary material for: Phenotypic and Functional Plasticity of CXCR6+ Peripheral Blood NK Cells
Source: Front Immunol. 2022 Jan 31;12:810080. doi: 10.3389/fimmu.2021.810080 (PMC8841448; doi:10.3389/fimmu.2021.810080)
Supplement: Supplementary file 1 [file DataSheet_1.pdf]

## Supplemental Figure 1

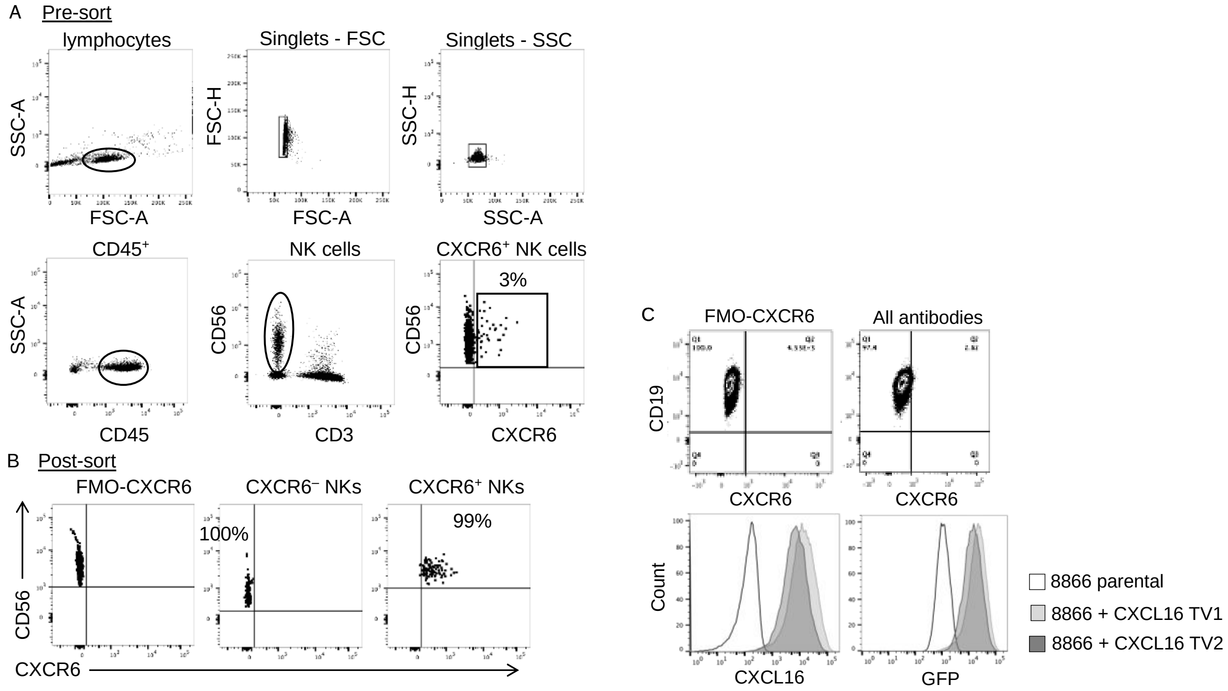


**Supplemental Figure 1. Gating strategy for sorting of CXCR6$^{+}$ and CXCR6$^{–}$ PB-NK cells and post-sort purity checks and CXCL16 expression on RPMI-8866 feeder cells. (A)** NK cells were enriched from human PB, stained with appropriate antibodies, and sorted on a FACS Aria II according to the gating strategy shown. **(B)** Dot plots are from one representative experiment of at least 15. CXCR6$^{+}$ and CXCR6$^{–}$ NK cells were gated using an FMO control (FMO-CXCR6). Post-sort purity checks of CXCR6$^{+}$ and CXCR6$^{–}$ NK cells routinely confirmed 94-100% pure populations. **(C)** CXCL16-expressing RPMI-8866 were used as feeders for the expansion of CXCR6$^{+}$ and CXCR6$^{–}$ NK cells in culture. CXCR6 expression in RPMI-8866 cells ranged from 0.3–2.62%. RPMI-8866 cells were transduced with lentiviral particles encoding either of two CXCL16 transcript variants (TVs) tagged with GFP. High expressing cells were sorted on a BD Aria II Cell Sorter. 97.5% of 8866-CXCL16-TV1 cells and 97.8% of 8866-CXCL16-TV2 cells expressed CXCL16 following transduction.

## Supplemental Figure 2

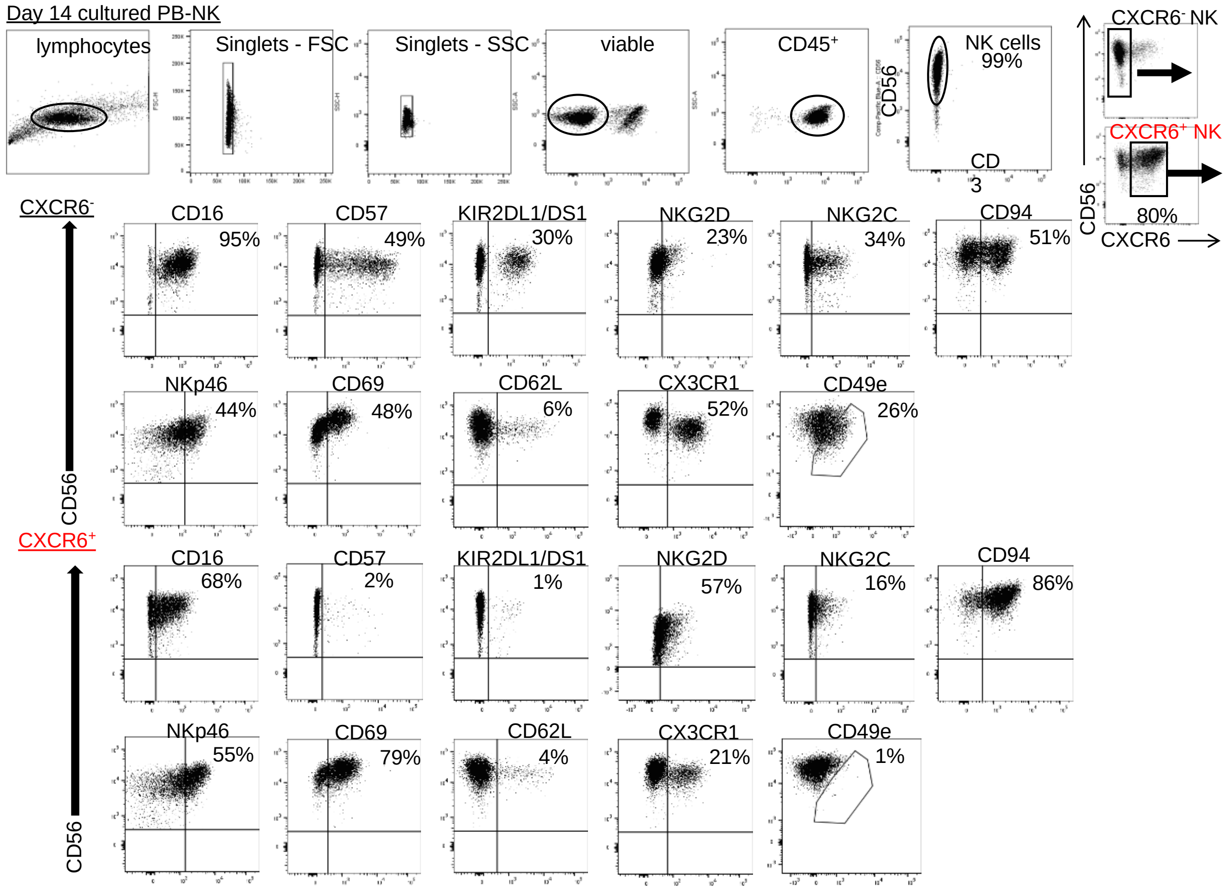


**Supplemental Figure 2. Gating strategy and representative flow plots of NK cell markers on day-14 cultured CXCR6$^{+}$ and CXCR6$^{–}$ PB-NK cells.** Since neither culture was 100% pure on day 14, CXCR6$^{–}$ NK within CXCR6$^{–}$ NK cell cultures were gated for marker expression, as were CXCR6$^{+}$ NK within CXCR6$^{+}$ NK cell cultures when determining percent expression. FMOs were used for gating positive populations.

# Supplemental Figure 3

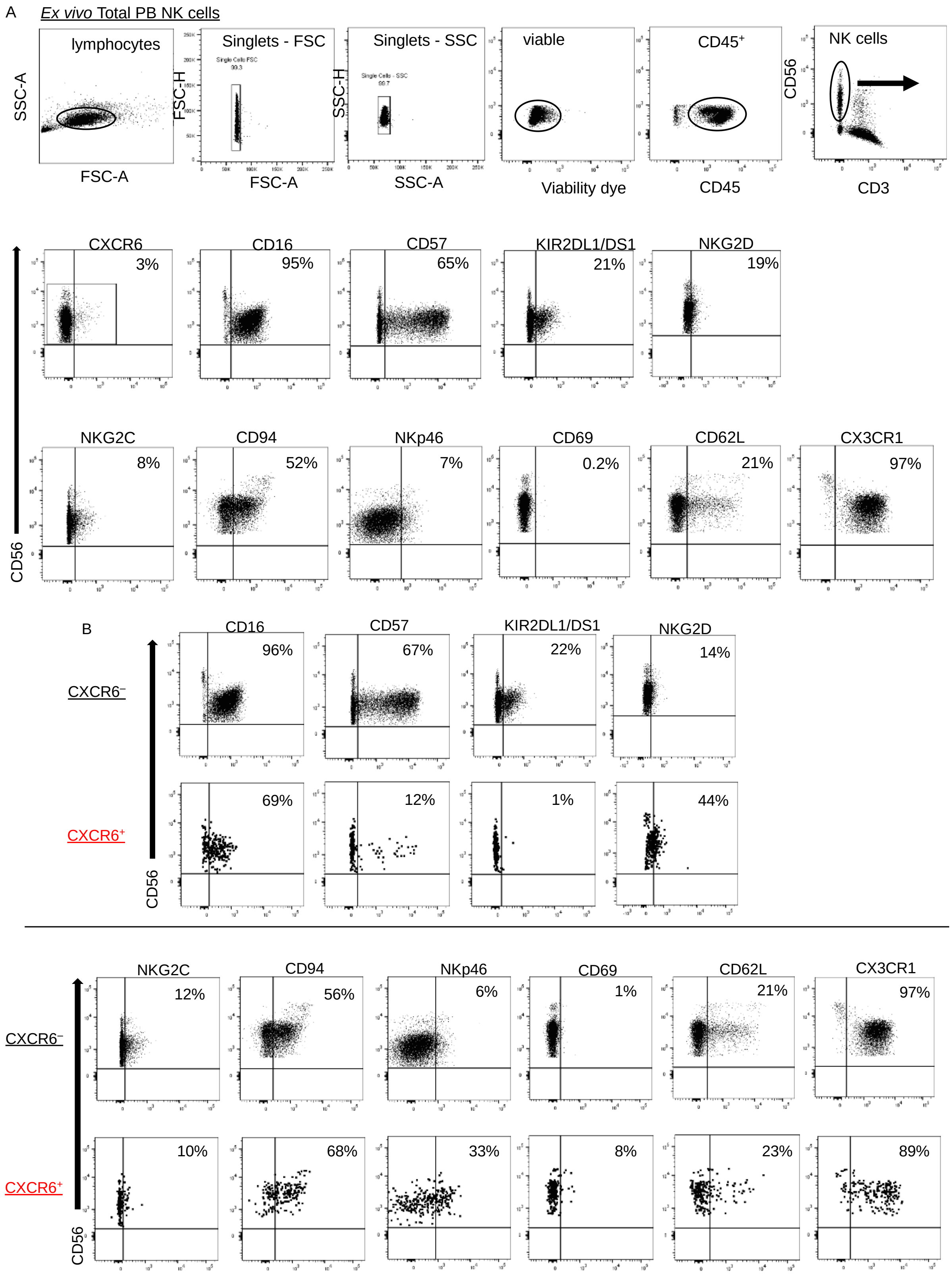

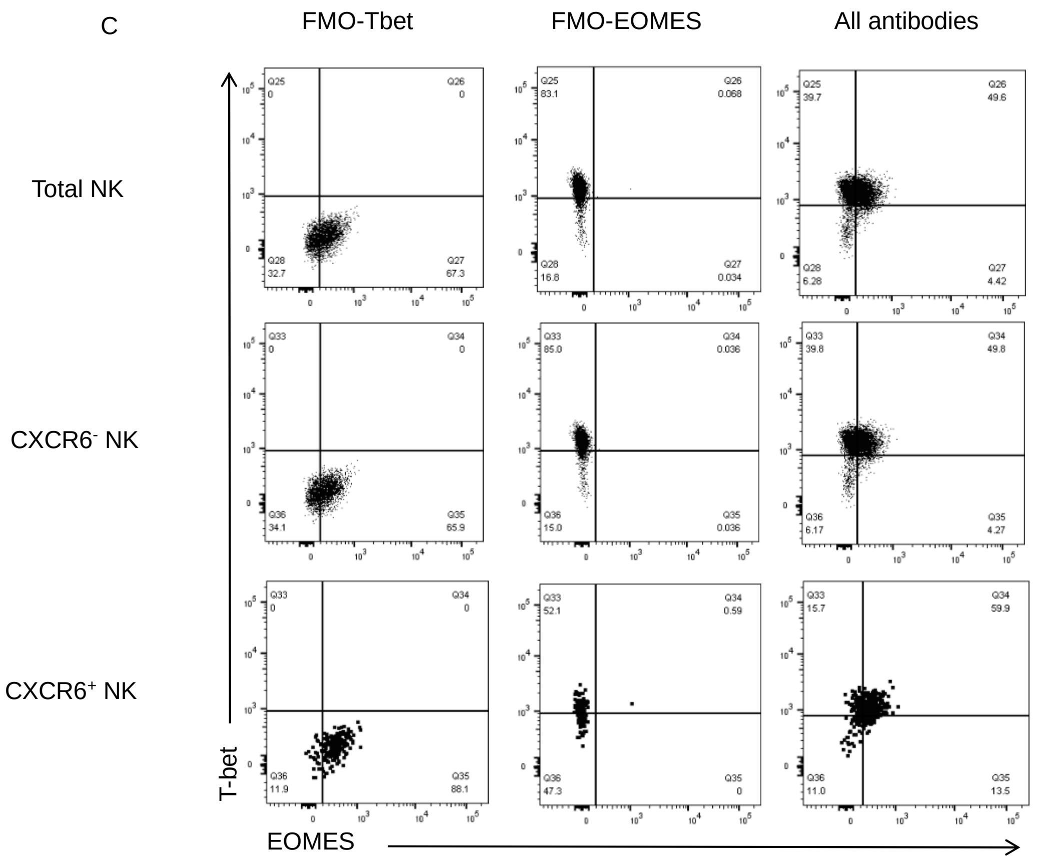


**Supplemental Figure 3.** Representative flow plots for (**A**) total *ex vivo* PB-NK cells and (**B**) CXCR6⁻ and CXCR6⁺ *ex vivo* PB-NK cells. Gating strategy and representative dot plots of NK cell markers are shown. FMOs were used for gating positive populations. (**C**) T-bet and EOMES FMOs delineate four subpopulations of T-bet/EOMES-expressing NK cells. T-bet and EOMES FMO controls with their respective all antibodies tubes in total, $CXCR6^-$, and $CXCR6^+$ NK cells.

## Supplemental Figure 4

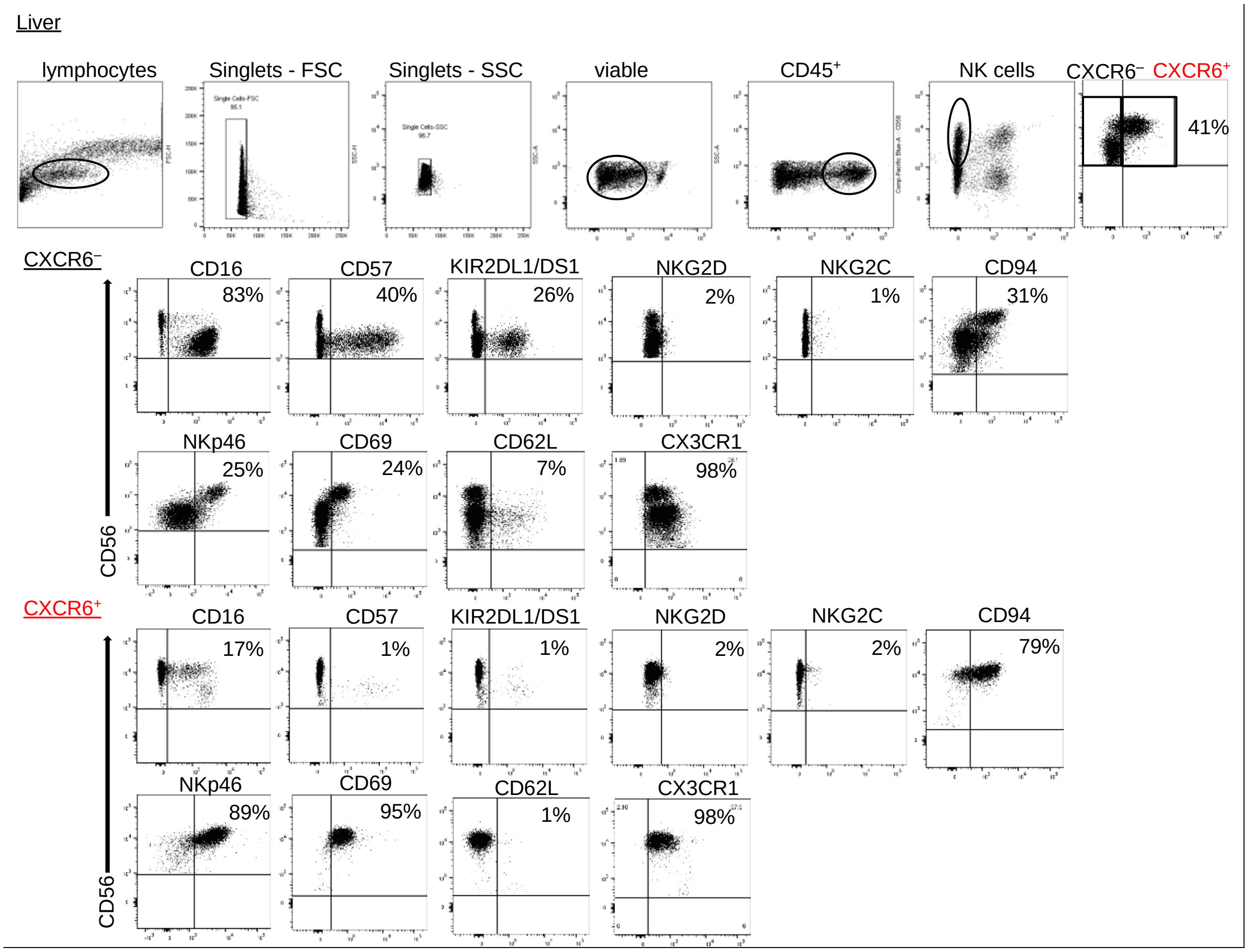


**Supplemental Figure 4. Gating strategy and representative flow plots for liver NK cells.** Liver NK cells were gated as shown, and NK cell markers on CXCR6$^+$ and CXCR6$^-$ NK cells were gated using FMO controls for each marker.

**Supplemental Figure 5**

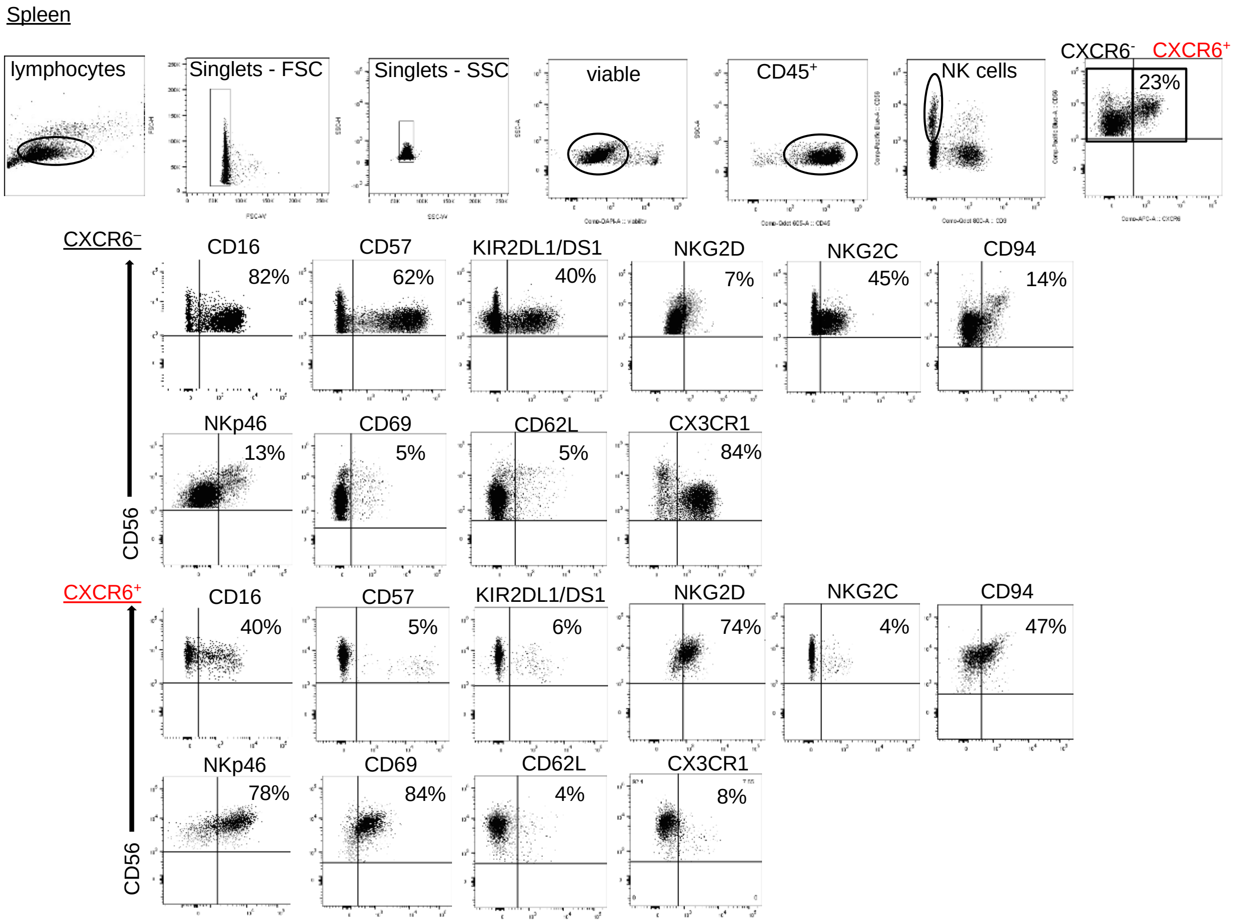


**Supplemental Figure 5. Gating strategy and representative flow plots for NK cell markers on spleen NK cells.** CXCR6$^+$ and CXCR6$^–$ NK were analyzed individually using FMO controls as a guide for positive populations.

**Supplemental Figure 6**

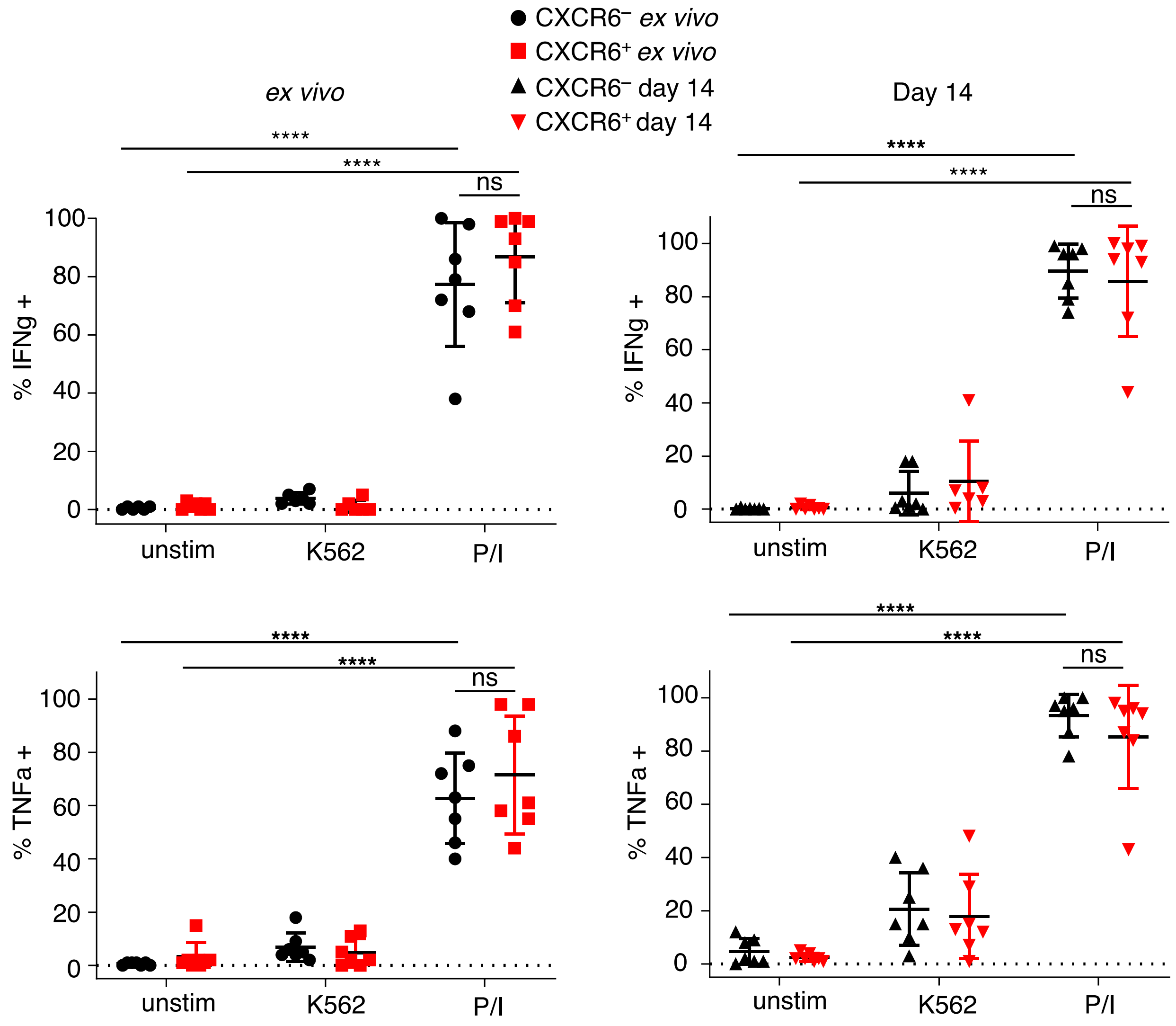


**Supplemental Figure 6. IFNg and TNF-a expression in *ex vivo* and day 14 CXCR6$^{+}$ and CXCR6$^{-}$ NK cells.** There is no significant difference in IFNg or TNF-a production in CXCR6$^{+}$ and CXCR6$^{-}$ NK cells following stimulation with K562 (1:1) or PMA/ionomycin in either *ex vivo* or day14-cultured NK (*n* = *7*).

**Supplemental Figure 7**

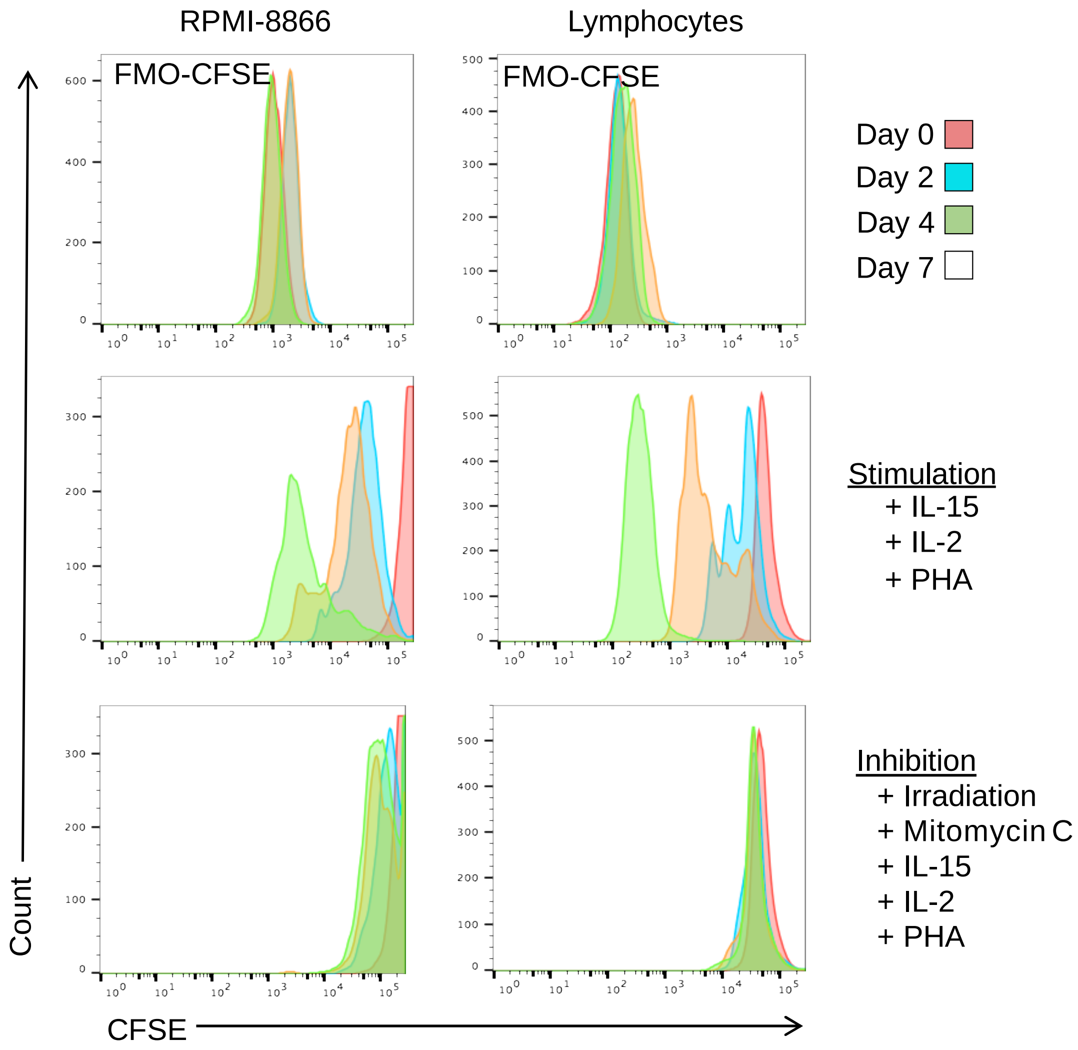


**Supplemental Figure 7. Irradiation and Mitomycin C effectively prevent feeder cell proliferation in CXCR6$^{+}$ and CXCR6$^{-}$ NK cell cultures.** Histograms are data from one representative experiment. 10,000 rads of x-ray irradiation and 10 µg/mL Mitomycin C effectively prevented proliferation of feeder cells (CXCL16-expressing RPMI-8866 B cells and lymphocytes).

**Supplemental Table 1. Percent CXCR6$^+$ NK cells in *ex vivo* PB, day 14 CXCR6$^-$ and CXCR6$^+$ NK cell cultures, liver, and spleen.**

| Source of NK cells | Mean % CXCR6$^+$ NK cells | n |
|---|---|---|
| Peripheral blood (ex vivo) | 7 | 33 |
| CXCR6$^-$ culture | 5 | 15 |
| CXCR6$^+$ culture | 74 | 15 |
| Liver | 34 | 9 |
| Spleen | 27 | 8 |

**Supplemental Table 2. Antibodies used for sorting**

| | fluorophore | marker | clone | company | catalog # |
|---|---|---|---|---|---|
| 1 | BV605 | CD45 | HI30 | Biolegend | 304042 |
| 2 | BV421 | CD56 | HCD56 | Biolegend | 318328 |
| 3 | BV711 | CD3 | OKT3 | Biolegend | 317328 |
| 4 | AF647 | CXCR6 | K041E5 | Biolegend | 356008 |

**Supplemental Table 3. Antibodies used for degranulation assays.**

| | fluorophore | marker | clone | company | catalog # |
|---|---|---|---|---|---|
| 1 | BV605 | CD45 | HI30 | Biolegend | 304042 |
| 2 | BV711 | CD3 | OKT3 | Biolegend | 317328 |
| 3 | PE-Dazzle594 | CD56 | HCD56 | Biolegend | 318348 |
| 4 | BV650 | CD16 | 3G8 | Biolegend | 302042 |
| 5 | AF647 | CXCR6 | K041E5 | Biolegend | 356008 |
| 6 | APC-Cy7 | CD20 | 2H7 | Biolegend | 302314 |
| 7 | BV786 | CD107a | H4A3 | BD Biosciences | 563869 |
| 8 | PE/Cy7 | TNFα | MAb11 | Biolegend | 502930 |
| 9 | AF700 | IFNγ | 4S.B3 | Biolegend | 502520 |
| 10 | PE | perforin | dG9 | Biolegend | 308106 |
| 11 | BV421 | Granzyme B | GB11 | Biolegend | 515408 |

**Supplemental Table 4. Antibodies used for phenotyping CXCR6$^+$ and CXCR6$^-$ NK cells, including transcription factors T-bet and EOMES.**

| | fluorophore | marker | clone | company | catalog # |
|---|---|---|---|---|---|
| 1 | BV605 | CD45 | HI30 | Biolegend | 304042 |
| 2 | BV785 | CD3 | OKT3 | Biolegend | 317330 |
| 3 | BV421 | CD56 | HCD56 | Biolegend | 318328 |
| 4 | APC/Cy7 | CD16 | B73.1 | Biolegend | 360710 |
| 5 | AF647 | CXCR6 | K041E5 | Biolegend | 356008 |
| 6 | AF700 | CD19 | HIB19 | Biolegend | 302226 |
| 7 | PE-Dazzle594 | CD57 | HNK-1 | Biolegend | 359620 |
| 9 | PE | NKG2C | 134591 | R&D Systems | FAB138P-100 |
| 10 | BV510 | NKG2D | 1D11 | Biolegend | 320816 |
| 11 | BV650 | NKp46 | 9E2/Nkp46 | BD Biosciences | 563230 |
| 12 | PE/Cy7 | KIR2DL1-DS1 | 11PB6 | Miltenyi | 130-099-891 |
| 14 | BV711 | Tbet | 4B10 | eBioscience | 644819 |
| 15 | PerCP-eFluor® 710 | EOMES | WD1928 | eBioscience | 46-4877-42 |

**Supplemental Table 5. Additional extracellular antibodies used for phenotyping** of $CXCR6^+$ and $CXCR6^-$ NK cells.

| | fluorophore | marker | clone | company | catalog # |
|---|---|---|---|---|---|
| 1 | BV605 | CD45 | HI30 | Biolegend | 304042 |
| 2 | BV711 | CD3 | OKT3 | Biolegend | 317328 |
| 3 | BV421 | CD56 | HCD56 | Biolegend | 318328 |
| 4 | APC/Cy7 | CD16 | B73.1 | Biolegend | 360710 |
| 5 | PE-Dazzle594 | CXCR6 | K041E5 | Biolegend | 356016 |
| 6 | FITC | CD49e | SKI-SAM-1 | Biolegend | 328008 |
| 7 | PE | CX3CR1 | 2A9-1 | Biolegend | 341604 |
| 8 | BV650 | CD69 | FN50 | Biolegend | 310934 |
| 9 | BV510 | NKG2D | 1D11 | Biolegend | 320816 |
| 10 | PE/Cy5 | CD62L | DREG-56 | Biolegend | 304808 |
| 11 | PerCP/Cy5.5 | CD94 | DX22 | Biolegend | 305514 |

## Supplemental Table 6. Statistical analysis for cytotoxicity assays (Figure 5A & B).

| Cell Orgin | Cells Compared | Cytotoxicity +/- IL-2 | | | | | | Statistics |
|---|---|---|---|---|---|---|---|---|
| | | 0.63 | 1.25 | 2.5 | 5 | 10 | 20 | |
| Ex Vivo | Within CXCR6+ | n.s. | n.s. | n.s. | n.s. | n.s. | n.s. | Two-way ANOVA with Tukey's Multiple Comparison Test |
| | Within CXCR6- | n.s. | n.s. | n.s. | n.s. | n.s. | n.s. | |
| Culture (Day 14) | Within CXCR6+ | n.s. | n.s. | n.s. | n.s. | n.s. | n.s. | |
| | Within CXCR6- | n.s. | n.s. | n.s. | n.s. | n.s. | n.s. | |

| Cell Orgin | Cells Compared | | Cytotoxicity | | | | | | Cytotoxicity + IL-2 | | | | | | Statistics |
|---|---|---|---|---|---|---|---|---|---|---|---|---|---|---|---|
| | | | 0.63 | 1.25 | 2.5 | 5 | 10 | 20 | 0.63 | 1.25 | 2.5 | 5 | 10 | 20 | |
| Ex Vivo | CXCR6+ | CXCR6- | n.s. | n.s. | * | 0.0511 | n.s. | n.s. | n.s. | n.s. | n.s. | n.s. | n.s. | n.s. | Two-way ANOVA with Tukey's Multiple Comparison Test |
| Culture (Day 14) | CXCR6+ | CXCR6- | n.s. | n.s. | n.s. | n.s. | n.s. | n.s. | n.s. | n.s. | n.s. | n.s. | n.s. | n.s. | |

## Statistical analysis for cytotoxicity assays (Figure 5C & D).

| Cell Orgin | Cells Compared | Cytotoxicity +/- IL-2 | | | | | | Statistics |
|---|---|---|---|---|---|---|---|---|
| | | 3.13 | 6.25 | 12.5 | 25 | 50 | 100 | |
| Liver | Within Liver | n.s. | n.s. | n.s. | n.s. | n.s. | | Two-way ANOVA with Tukey's Multiple Comparison Test |
| | Within PBMC | n.s. | n.s. | n.s. | n.s. | n.s. | | |
| Spleen | Within Spleen | n.s. | n.s. | n.s. | n.s. | n.s. | n.s. | |
| | Within PBMC | n.s. | n.s. | n.s. | n.s. | n.s. | n.s. | |

| Cell Orgin | Cells Compared | | Cytotoxicity | | | | | | Cytotoxicity + IL-2 | | | | | | Statistics |
|---|---|---|---|---|---|---|---|---|---|---|---|---|---|---|---|
| | | | 3.13 | 6.25 | 12.5 | 25 | 50 | 100 | 3.13 | 6.25 | 12.5 | 25 | 50 | 100 | |
| Liver | Liver | PBMC | n.s. | * | *** | **** | *** | | n.s. | ** | **** | **** | **** | | Two-way ANOVA with Tukey's Multiple Comparison Test |
| Spleen | Spleen | PBMC | n.s. | n.s. | n.s. | n.s. | n.s. | * | n.s. | n.s. | * | ** | *** | **** | |

## Supplemental Table 7. Antibodies for pSTAT5 flow cytometry

| | fluorophore | marker | clone | company | catalog # |
|---|---|---|---|---|---|
| 1 | BV605 | CD45 | HI30 | Biolegend | 304042 |
| 2 | BV421 | CD56 | HCD56 | Biolegend | 318334 |
| 3 | BV711 | CD3 | OKT3 | Biolegend | 317328 |
| 4 | AF647 | P-STAT5 (Y694) | 47/Stat5(pY694) | BD Biosciences | 612599 |

## Supplemental Table 8. Statistical analysis for ADCC assays (Figure 6A & B).

| Cell Orgin | Cells Compared | ADCC +/- rit | | | | | | Statistics |
|---|---|---|---|---|---|---|---|---|
| | | 0.63 | 1.25 | 2.5 | 5 | 10 | 20 | |
| Ex Vivo | Within CXCR6+ | n.s. | n.s. | * | **** | **** | **** | Two-way ANOVA with Tukey's Multiple Comparison Test |
| | Within CXCR6- | n.s. | n.s. | ** | **** | **** | **** | |
| Culture (Day 14) | Within CXCR6+ | n.s. | n.s. | n.s. | * | ** | ** | |
| | Within CXCR6- | n.s. | n.s. | n.s. | n.s. | n.s. | n.s. | |

| Cell Origin | Cells Compared | ADCC | | | | | | ADCC + rit | | | | | | Statistics |
|---|---|---|---|---|---|---|---|---|---|---|---|---|---|---|
| | | 0.63 | 1.25 | 2.5 | 5 | 10 | 20 | 0.63 | 1.25 | 2.5 | 5 | 10 | 20 | |
| ex vivo | CXCR6+ vs CXCR6- | n.s. | n.s. | n.s. | n.s. | n.s. | n.s. | n.s. | n.s. | n.s. | n.s. | n.s. | n.s. | Two-way ANOVA with Tukey's Multiple Comparison Test |
| 14 day culture | CXCR6+ vs CXCR6- | n.s. | n.s. | n.s. | n.s. | n.s. | n.s. | n.s. | n.s. | n.s. | n.s. | n.s. | n.s. | |

## Statistical analysis for ADCC assays (Figure 6C & D).

| Cells Compared | ADCC +/- rit | | | | | | Statistics |
|---|---|---|---|---|---|---|---|
| | 3.13 | 6.25 | 12.5 | 25 | 50 | 100 | |
| Within Liver | n.s. | n.s. | n.s. | * | * | | Two-way ANOVA with Tukey's Multiple Comparison Test |
| Within PBMC | n.s. | n.s. | n.s. | *** | **** | | |
| Within Spleen | n.s. | n.s. | n.s. | n.s. | n.s. | n.s. | |
| Within PBMC | n.s. | n.s. | n.s. | *** | **** | **** | |

| Cells Compared | | ADCC | | | | | | ADCC +/- rit | | | | | | Statistics |
|---|---|---|---|---|---|---|---|---|---|---|---|---|---|---|
| | | 3.13 | 6.25 | 12.5 | 25 | 50 | 100 | 3.13 | 6.25 | 12.5 | 25 | 50 | 100 | |
| Liver | PBMC | n.s. | n.s. | n.s. | n.s. | n.s. | | n.s. | n.s. | n.s. | n.s. | * | | Two-way ANOVA with Tukey's Multiple Comparison Test |
| Spleen | PBMC | n.s. | n.s. | n.s. | n.s. | n.s. | n.s. | n.s. | n.s. | n.s. | *** | **** | **** | |
